# Unprecedented reactions: from epichlorohydrin to epoxyglycidyl substituted divinyl ether and its conversion into epoxyglycidyl propargyl ether

**DOI:** 10.1038/srep14231

**Published:** 2015-09-18

**Authors:** Yiwu Yao, Zheng Li, Yatao Qiu, Jinhong Bai, Jinyue Su, Dayong Zhang, Sheng Jiang

**Affiliations:** 1 Laboratory of Medicinal Chemistry, Guangzhou Institute of Biomedicine and Health, The Chinese Academy of Sciences, Guangzhou 510530, China; 2 The Methodist Hospital Research Institute, Houston, Texas 77030, United States; 3 State Key Laboratory of Natural Medicines and Center of Drug Discovery, China Pharmaceutical University, Nanjing 210009, China

## Abstract

The reaction of epichlorohydrin with concentrated sodium hydroxide in hexane under phase transfer conditions has surprisingly led to the formation of the symmetrical di(3-epoxyglycidyl-1-propenyl) ether **1** which contains both nucleophilic and electrophilic moieties. When it was reacted with n-butyllithium, intermediate **1** once again surprisingly generated epoxyglycidyl propargyl ether, which was further reacted *in situ* with a variety of benzaldehydes to furnish the corresponding substituted propargylic alcohols in good yields. While the reaction is operationally simple, it provides a powerful method for the synthesis of the important products from commodity materials such as epichlorohydrin. Moreover, these reactions may have revealed that some fundamental properties of the hydroxide anion in those once thought straightforward reactions are not well understood. A careful analysis of the experimental data suggests that an unprecedented concerted elimination of the epoxyglycidyl ether with sodium hydroxide may be operative and an alpha deprotonation followed by alpha elimination of the di(3-epoxyglycidyl-1-propenyl) ether with alkyllithium may have been involved.

Divinyl ethers such as **1** are important raw materials, primarily for the production of vinyl polymeric materials containing oxygen bridges which are expected to be biodegradable in nature^1^,^2^,^3^. Divinyl ether itself has rather unique thermodynamic and spectroscopic properties^4^,^5^,^6^,^7^,^8^ and it was first prepared by the exhaustive methylation of morpholine by Knorr and Matthes in 1899^9^. Because the synthetic reaction was performed under harsh conditions in low yields, much efforts have been devoted to the efficient preparation of those divinyl ethers. For example, dehydrohalogenation of the corresponding β,β’-dihalo ethers at elevated temperatures^10^, isomerization of diallyl ethers using palladium on carbon^11^, decomposition of 5,5-disubstituted-3-nitrosooxazolidones^12^, and Wittig olefination of aldehydes and ketones^13^.

Symmetrical di(3-epoxyglycidyl-1-propenyl) ethers containing both the divinyl ether and the epoxide moieties **1** (Fig. 1) have both nucleophilic and electrophilic sites of reaction and may readily be converted into a variety of useful monomers for the synthesis of environmentally benign and biodegradable polymeric materials^14^. However, di(3-epoxyglycidyl-1-propenyl) ethers are difficult to synthesize using the existing methods due to the presence of both electrophilic and nucleophilic functional groups associated with those molecules. Therefore, an efficient method that is economical and operationally simple for the synthesis of such intermediates containing both the divinyl ether and the epoxyglycidyl moieties starting from commodity materials is highly desirable.

In the study described below, we disclose a novel reaction of epichlorohydrin that has led to the formation of the highly desired di(3-epoxyglycidyl-1-propenyl) ether **1**. Upon reaction with n-butyllithium, the epoxyglycidyl substituted divinyl ether was transformed into a terminal acetylene which was reacted *in situ* with aromatic aldehydes to generate the epoxyglycidyl propargyl alcohols.

## Results

In an initial experiment, slow addition of NaOH powder (100 mmol, 5.0 eq) into a mixture containing epichlorohydrin (20 mmol, 1.0 eq), hexane (17 mL), an aqueous NaOH solution (30%, 4 mL), and a catalytic amount of tetrabutylammonium hydrogensulfate (0.5 mmol, 2.5 mol%) at room temperature afforded a pure product in 44% yield after flash chromatography (Table 1, entry 1). The ^1^H and ^13^C NMR data analyses indicated that the structure of the product contained both the epoxide and the vinyl ether functional groups. The H-H COSY and C-H COSY spectroscopic data (see supporting information) showed that the structure of the product was **1**. During the optimization of the reaction conditions, it was found that a variety of bases (solid) could be utilized in the reaction and NaOH provided the highest yield (60%, entry 2). Using NaOH as the base and hexane as the solvent, reactions employing other concentrations of aqueous NaOH solutions, including 40% and 50% were explored and the highest yield was obtained when a 50% NaOH solution was used (Table 1, entries 1–3). The organic solvent was also found to play an important role in this process (entries 8–11) and hexane was found to be the most suitable solvent for this reaction (entry 2). The yields of the desired product was also dependent on the amount of the phase transfer catalyst (e.g., Bu_4_NHSO_4_), without which the reaction did not occur (entries 3 and 12–14). In addition, when the temperature was raised to around 40 ^o^C or reduced to near 10 ^o^C, the yields of the process decreased to 46% and 34%, respectively (entries 6 and 7). Moreover, the yields of the reaction increased with time but started to decrease after 12 hours. These results of the preliminary optimization process showed that a better reaction condition was to use epichlorohydrin (20 mmol, 1.0 eq.) in hexane (17 mL) together with Bu_4_NHSO_4_ (0.5 mmol, 2.5 mol%), solid NaOH (100 mmol, 5 eq.) and a 50% aqueous NaOH solution (100 mmol, 5 eq.) for 12 h at room temperature (28 °C).

**Figure 2:**
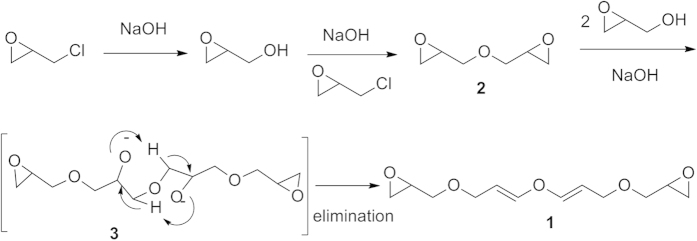
The proposed mechanism for the formation of 1.

The structure of the reaction product was surprising since the potential anionic polymerization or epoxide hydrolysis did not substantiate under the reaction conditions, nor did the epoxide functional group be hydrolyzed. Thus, sodium hydroxide acted only as a strong base. A possible mechanism for the formation of **1** was formulated in Fig. 2. Epichlorohydrin was transformed into the epoxypropanol intermediate, which was further reacted with epichlorohydrin to form diepoxyglycidyl ether **2**. In the presence of a strong base, intermediate **2** readily reacted with 2.0 equiv of epoxypropanol to afford intermediate **3**, which underwent a simultaneous *in situ* elimination possibly through a concerted intramolecular proton abstraction-elimination process via a six-membered ring transition state. If the elimination were not concerted, it would be more likely to give rise to glycidyl 3-epoxyglycidyl-1-propenyl ether, instead of the di(3-epoxyglycidyl-1-propenyl) ether. Since sodium hydroxide was in excess, the ring opening of the epoxide of the product was expected. However, no epoxide opening product was observed, which suggested that the hydroxide anion did not have nucleophilicity under the reaction conditions, it only acted as a base. In addition, no hexamerization or octamerization product from epichlorohydrin was observed. To the best of our knowledge, this is the first example of the reaction of epichlorohydrin leading to the formation of glycidyl 3-epoxyglycidyl-1-propenyl ether.

With di(3-epoxyglycidyl-1-propenyl) ether **1** in hand, we focused on the reaction of **1** with n-butyllithium, trying to obtain the addition product of n-butyllithium to the epoxide. However, the results were surprising. The epoxide functional groups remained intact while the di(3-epoxyglycidyl-1-propenyl) ether decomposed into two molecules of epoxyglycidyl propargyl ether. The n-butyl anion only showed its basicity, no nucleophilicity was indicated. The epoxyglycidyl propargyl ether intermediate was then trapped *in situ* with benzaldehyde. As a model reaction for the optimization of the reaction conditions, a brief study of the ratio between compound **1**, n-BuLi and benzaldehyde was carried out. As shown in Table 2, it was found that when the ratio between **1**, n-BuLi and benzaldehyde was 1:3:3, the reaction produced good yields (entries 1–7). The reaction time was also evaluated in the same reaction system. The yields increased with time but began to decrease after 5 hours (entries 3, 8–10). The solvent also played an important role but the best solvent was found to be THF (tetrahydrofuran) amongst those being tested. These results showed that the optimized conditions for the reaction of **1** with n-BuLi and benzaldehyde involved the use of **1** (1.0 equiv), n-BuLi (3.0 equiv, 2.5 N concentration) and benzaldehyde (3.0 equiv) in THF at −78 ^o^C under an argon atmosphere. The reaction temperature was maintained at −78 ^o^C before the addition of the benzaldehydes.

**Figure 3:**
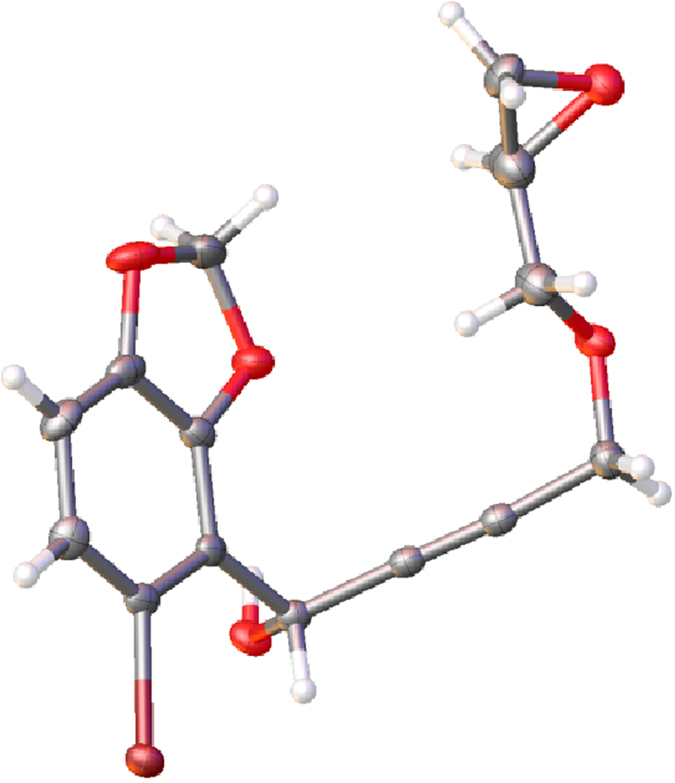
The crystal structure of 5b confirmed by X-ray crystallographic data analysis.

When the epoxyglycidyl propargyl ether intermediate from the reaction of **1** with nBuLi was reacted with 5-bromobenzo[d] [1,3]dioxole-4-carbaldehyde, it afforded the corresponding product **5b** in 47% yield in the crystalline form. Based on the X-ray crystallographic data analysis, the structure of **5b** is shown in Fig. 3 (The X-ray crystallographic data were deposited at the Cambridge Crystallographic Data Centre and the file number is CCDC 982788). The trapping reaction was then extended to a variety of aromatic aldehydes and all have provided good yields (see supporting information).

**Figure 4:**
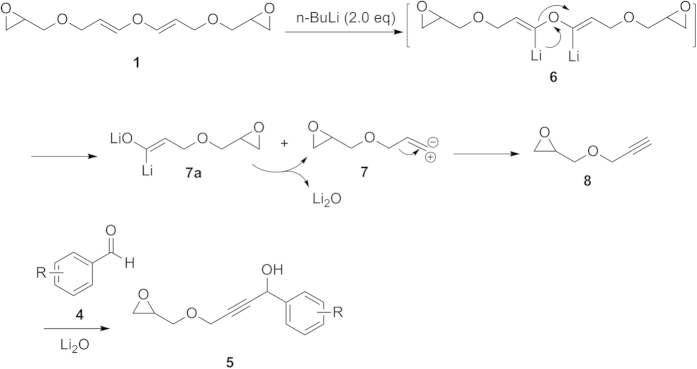
The proposed mechanism for intermediate 1 with nBuLi.

On the basis of the above results, a tentative reaction mechanism for the formation of **5** is proposed (Fig. 4) though other mechanisms may also be operative. The divinyl ether **1** was initially dilithiated to form **6**, which decomposed to generate a carbenoid intermediate **7** and the lithium alkenoxide **7a**. The latter further decomposed to form intermediate **7** and lithium oxide. Intermediate **7** simultaneously rearranged to afford epoxyglycidyl propargyl ether **8**, which reacted with benzaldehydes in the presence of a strong base (either Li_2_O or nBuLi) to give the addition product **5**. To the best of our knowledge, the transformation of di(3-epoxyglycidyl-1-propenyl) ether **1** into epoxyglycidyl propargyl ether **8** is the first example of this type of reaction.

In summary, we have discovered a highly efficient methodology that solely uses epichlorohydrin and NaOH at room temperature to generate the symmetrical di(3-epoxyglycidyl-1-propenyl) ether **1**. When the latter was reacted with nBuLi, it generated epoxyglycidyl propargyl ether **8**. To the best of our knowledge, these two reactions have not been reported before. These two transformations have not only constituted important while convenient methods for the conversion of epichlorohydrin into di(3-epoxyglycidyl-1-propenyl) ether and its conversion into epoxyglycidyl propargyl ether, but they have also enabled us to better understand the properties of hydroxide anion and butyl carbanion in aprotic solvents.

## Methods

A standard procedure for the first reaction: The solution of NaOH (4 g, 100 mmol) in H_2_O (4 mL) was added into the mixture of epichlorohydrin (1.85 g, 20 mmol), tetrabutylammonium hydrogen sulfate (0.17 mg, 0.5 mmol) and n-hexane (17 mL) at 28 ^o^C. The mixture was stirred for 30 min, followed by addition of NaOH (4 g, 100 mmol), and then stirred at room temperature for 12 h. The upper layer was separated, and the residue was diluted with water carefully at 0 ^o^C, and then extracted with hexane (3 × 15 mL). The combined organic layers were washed with saturated brine, dried with anhydrous Na_2_SO_4_ and evaporated. The residue was purified by flash chromatography on silica gel to provide**1** as colorless oil (0.73 g, 60%).

A standard procedure for the second reaction: To a stirred solution of the compound **1** (484 mg, 2.0 mmol) in dry THF (20 mL) was added n-BuLi (2.4 mL, 6.0 mmol, 2.5 M in hexane) at −78 ^o^C. The mixture was stirred for 1.0 h, followed by addition of benzaldehyde (6.0 mmol) in dry THF (1 mL). After further stirring for 3 h at the same temperature, the reaction mixture was quenched by saturated ammonium chloride (5 mL). The residue was extracted with ethyl acetate (3 × 15 mL). The combined organic layers were washed with saturated brine, dried with anhydrous Na_2_SO_4_ and evaporated. The residue was purified by chromatography on silica gel to afford the product.

The X-ray crystallographic coordinates for the structures of compound **5b** (CCDC 982788) was deposited with the Cambridge Crystallographic Data Center. These data can be obtained free of charge at www.ccdc.cam.ac.uk/retrieving.html.

## Additional Information

**How to cite this article**: Yao, Y. *et al.* Unprecedented reactions: from epichlorohydrin to epoxyglycidyl substituted divinyl ether and its conversion into epoxyglycidyl propargyl ether. *Sci. Rep.* **5**, 14231; doi: 10.1038/srep14231 (2015).

## Supplementary Material

Supplementary Information

## Figures and Tables

**Figure 1:**
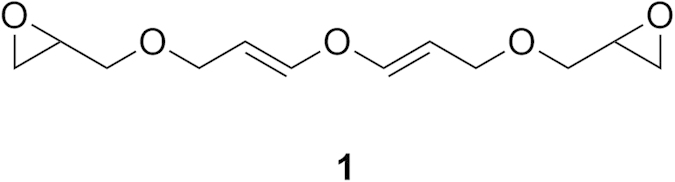
Structure of a symmetrical epoxyglycidyl substituted divinyl ether 1.

**Table 1:**
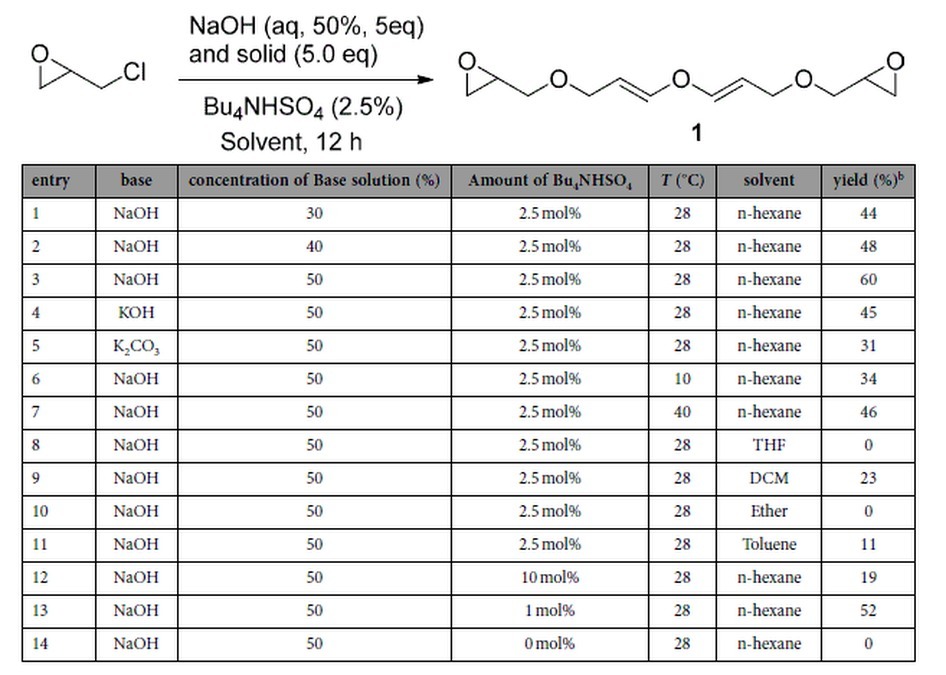
Optimization of the reaction conditions leading to the formation of **1**.^a^

^a^1 (20.0 mmol), NaOH solution (100.0 mmol), solid base (100.0 mmol), solvent (17 mL), 28 ^o^C, 12 h.

^b^Isolated yield.

**Table 2:**
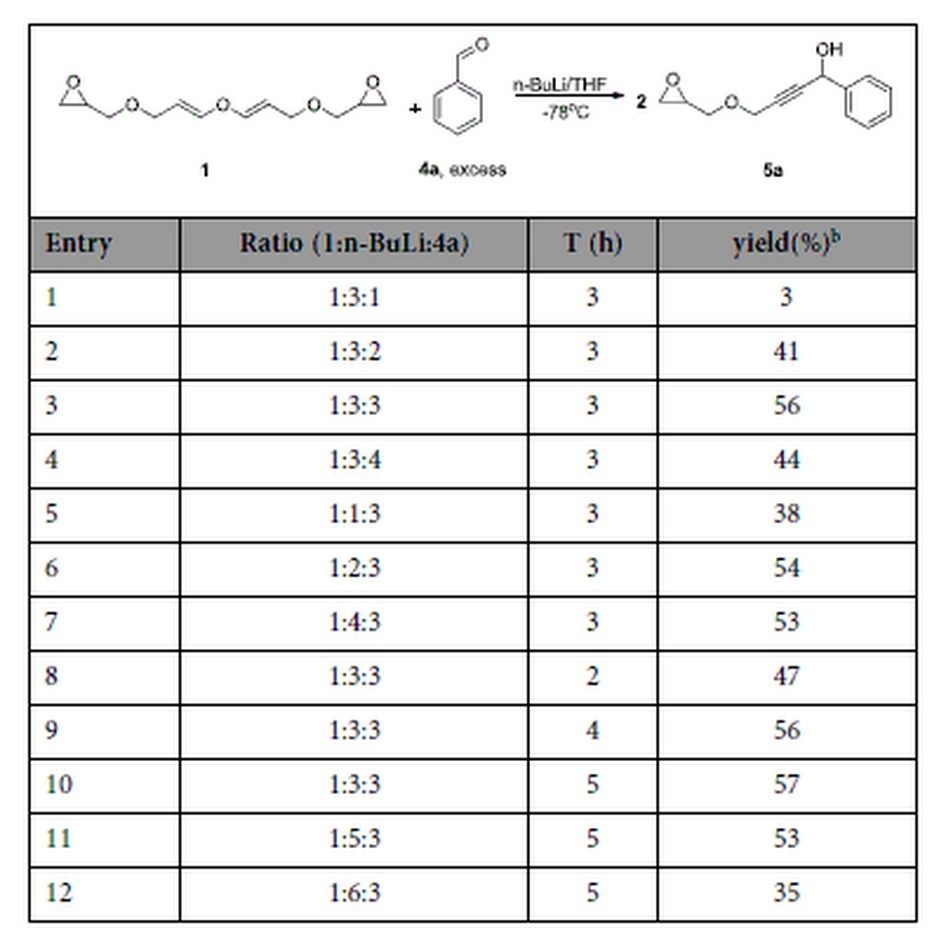
Optimization of reaction conditions for 1 with nBuLi and trapping the intermediate product with benzaldehyde.^a^

^a^Reaction conditions: **1** (1.0 mmol), n-BuLi (3.0 mmol) and benzaldehyde **4a** (3.0 mmol) in 10 mL of THF at −78 ^o^C under argon.

^b^Isolated yield.
