# Supplementary material for: Unprecedented reactions: from epichlorohydrin to epoxyglycidyl substituted divinyl ether and its conversion into epoxyglycidyl propargyl ether
Source: Sci Rep. 2015 Sep 18;5:14231. doi: 10.1038/srep14231 (PMC4585610; doi:10.1038/srep14231)
Supplement: Supplementary Information [file srep14231-s1.pdf]

Supporting Information

Unprecedented reactions: from epichlorohydrin to epoxyglycidyl substituted divinyl ether and its conversion into epoxyglycidyl propargyl ether

Yiwu Yao[1], Zheng Li[2], Yatao Qiu[1], Jinhong Bai[1], Jinyue Su[1,3], Dayong Zhang[3], Sheng Jiang[1,]*

[1]*Laboratory of Medicinal Chemistry, Guangzhou Institute of Biomedicine and Health, The Chinese Academy of Sciences, Guangzhou 510530, China*;

[2]*The Methodist Hospital Research Institute, Houston, Texas 77030, United States;*

[3]*State Key Laboratory of Natural Medicines and Center of Drug Discovery, China Pharmaceutical University, Nanjing 210009, China*

* *corresponding.author:jiang_sheng@gibh.ac.cn*

List of Contents

1. General Experimental Procedures and Spectroscopic Data of Compounds (S2)

2. Copies of ^{1}H and ^{13}C NMR of products (S16)

3. Crystallographic Data (S40)

1. General Experimental Procedures and Spectroscopic Data of Compounds

General Procedures: ^{1}H NMR and ^{13}C NMR spectra were recorded on Bruker Avance ARX- 400. Mass spectra were performed on Kompact Axima-CFR MALDI mass spectrometers. Optical rotations were recorded on a PerkinElmer 341 polarimeter. Anhydrous solvents were obtained as follows: THF and diethylether by distillation from sodium and benzophenone; dichloromethane fromCaH_2. All other solvents were reagent grade. All moisture sensitive reactions were carriedout in flame dried flask under argon atmosphere.

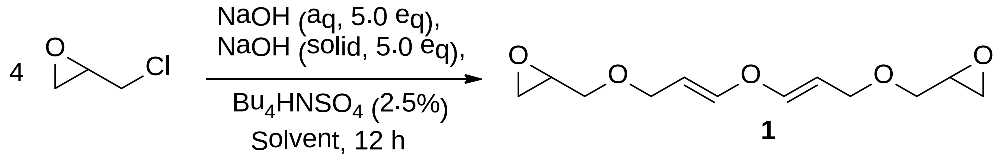

1

2-(((E)-3-((E)-3-(oxiran-2-ylmethoxy)prop-1-enyloxy)allyloxy)methyl)oxirane (**1**): The solution of NaOH (4g, 100 mmol) in H_2O (4 mL) was added into the mixture of epichlorohydrin (1.85g, 20 mmol), tetrabutylammonium hydrogen sulfate (0.17mg, 0.5 mmol) and n-hexane (17mL) at 28 ^{o}C. The mixture was stirred for 30 min, followed by addition of NaOH (4g, 100 mmol), and then stirred at room temperature for 12 h. The mixture was diluted with water carefully at 0 ^{o}C, and then extracted with hexane (3x 15 mL). The combined organic layers were washed with saturated brine, dried with anhydrous Na_2SO_4 and evaporated. The residue was purified by flash chromatography on silica gel to provide **1** as colorless oil (0.73g, 60%). ^{1}H NMR (400 MHz, $CDCl_3$) δ 6.24 (dt, *J*= 13.2, 1.6 Hz, 2H), 6.03-5.97 (m, 2H), 4.07 – 3.97 (m, 4H), 3.74 (dd, *J* = 11.6, 6.8 Hz, 2H), 3.35 (m,2H), 3.15-3.11 (m, 2H), 2.79-2.77 (m, 2H),

2.63-2.58 (m, 1H); ^{13}C NMR (125 MHz, $CDCl_3$) δ 129.5, 121.4, 70.8, 69.2, 50.6, 44.0. HRMS (ESI): calcd for $C_{12}H_{18}O_5$ $[M+H]^+$ 243.1232, found 243.1229.

General procedure B: the synthesis of Propargylic Alcohols with Oxirane

To a stirred solution of the compound **1** (484 mg, 2.0 mmol) in dry THF (20mL) was added n-BuLi (2.4 mL, 6.0 mmol, 2.5 M in hexane) at -78°C. The mixture was stirred for 1h, followed by addition of benzaldehyde (6.0 mmol) in dry THF (1 mL). After further stirring for 3h at the same temperature, the reaction mixture was quenched by saturated ammonium chloride (5 mL). The residue was extracted with ethyl acetate (3×15 mL). The combined organic layers were washed with saturated brine, dried with anhydrous Na_2SO_4 and evaporated. The residue was purified by chromatography on silica gel to afford the product.

Table S1. Reaction of **1** with various substituted benzaldehydes.[a]

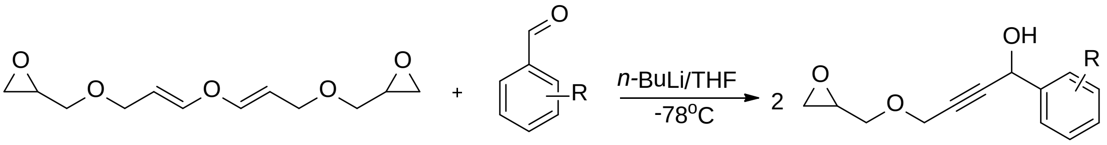

1 **4** **5**

Entry	Product	yield ^{b} (%)	Entry	Product	yield ^{b} (%)
1	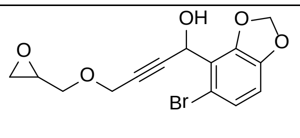 **5b**	47	11	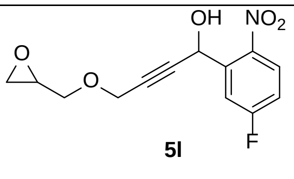 **5l**	50
2	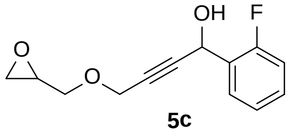 **5c**	54	12	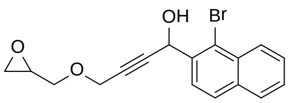 **5m**	56

3	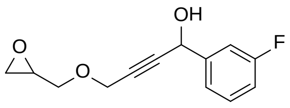 5d	61	13	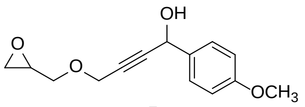 5n	57
4	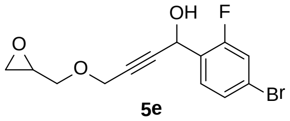 5e	61	14	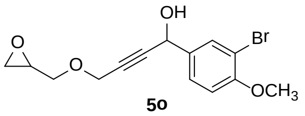 5o	52
5	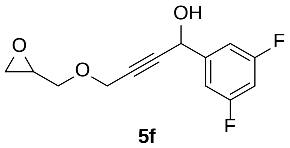 5f	54	15	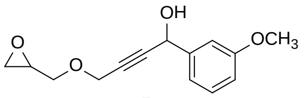 5p	50
6	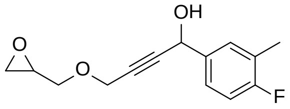 5g	69	16	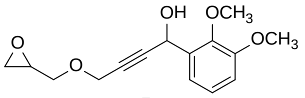 5q	55
7	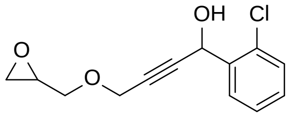 5h	59	17	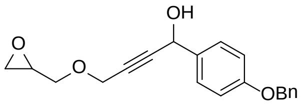 5r	38
8	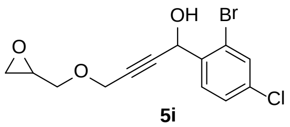 5i	58	18	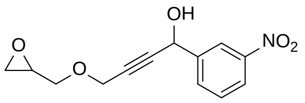 5s	49
9	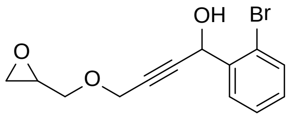 5j	44	19	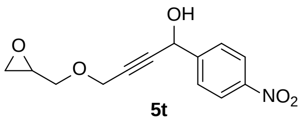 5t	39
10	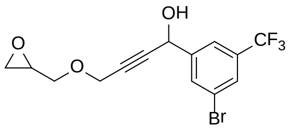 5k	45	20	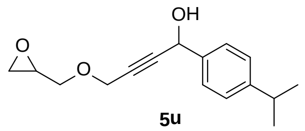 5u	44

[a] Reaction conditions: **3** (2.0 mmol), n-BuLi (6.0 mmol, 2.5 M in hexane) and substituted benzaldehyde (6.0 mmol) in 21.0 mL of THF at -78 °C under argon. [b] Isolated yield.

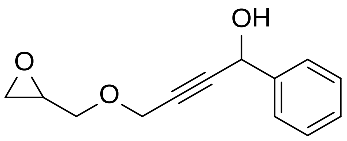

5a

4-(oxiran-2-ylmethoxy)-1-phenylbut-2-yn-1-ol (5a)

Following procedure A, **1** (970 mg, 4.0 mmol) was allowed to react with benzaldehyde (1.27 g, 12.0 mmol) at -78°C. The crude brown oil was purified by flash chromatography on silica gel to provide **5a** as a colorless liquid (57%). ^{1}H NMR (400 MHz, $CDCl_3$) δ 7.52 (d, *J* = 7.3 Hz, 2H), 7.43 – 7.28 (m, 3H), 5.50 (d, *J* = 5.2 Hz, 1H), 4.30 (t, *J* = 1.9 Hz, 2H), 3.83 (dd, *J* = 11.4, 3.0 Hz, 1H), 3.47 (dd, *J* = 11.4, 5.9 Hz, 1H), 3.21 – 3.12 (m, 1H), 2.79 (ddd, *J* = 5.0, 4.1, 0.8 Hz, 1H), 2.62 (dd, *J* = 5.0, 2.7 Hz, 1H), 2.57 (d, *J* = 5.8 Hz, 1H); ^{13}C NMR (125 MHz, $CDCl_3$) δ 140.4, 128.6, 128.4, 126.5, 86.5, 82.2, 70.4, 64.5, 58.8, 50.6, 44.3; HRMS (ESI): calcd for $C_{13}H_{14}O_3$ $[M+Na]^+$ 362.9844, found 362.9845.

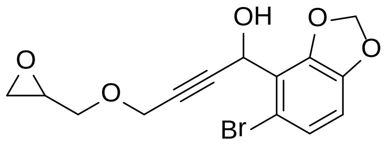

5b

1-(5-bromobenzo[d][1,3]dioxol-4-yl)-4-(oxiran-2-ylmethoxy)but-2-yn-1-ol (5b)

Following procedure A, **1** (484 mg, 2.0 mmol) was allowed to react with 4-fluoro-3-methylbenzaldehyde (1.36 g, 6.0 mmol) at -78°C. The crude brown oil was purified by flash chromatography on silica gel to provide **5b** as a white solid (47%). ^{1}H NMR (400 MHz, $CDCl_3$) δ 7.03 (d, *J* = 8.3 Hz, 1H), 6.67 (d, *J* = 8.3 Hz, 1H), 6.06 (s, 2H), 5.80 (d, *J* = 9.5 Hz, 1H), 4.27 (dd, *J* = 3.3, 1.8 Hz, 2H), 3.84 – 3.73 (m, 1H), 3.48 (ddd, *J* = 11.4, 5.8, 3.7 Hz, 1H), 3.19 – 3.11 (m, 1H), 3.05 (d, *J* = 9.5 Hz, 1H), 2.80 (dd, *J* = 5.0, 4.1 Hz, 1H), 2.65 – 2.57 (m, 1H); ^{13}C NMR (125 MHz, $CDCl_3$) δ 147.6, 146.1, 125.8, 122.2, 112.5, 109.4, 102.2, 84.8, 81.6, 70.3, 61.5, 58.7, 50.5, 44.4; HRMS (ESI): calcd for $C_{14}H_{13}BrO_5$ $[M+Na]^+$ 362.9844, found 362.9846.

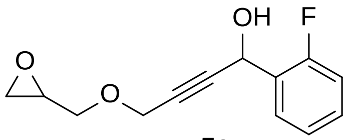

5c

1-(2-fluorophenyl)-4-(oxiran-2-ylmethoxy)but-2-yn-1-ol (5c)

Following procedure A, **1** (484 mg, 2.0 mmol) was allowed to react with 2-fluorobenzaldehyde (744 mg, 6.0 mmol) at -78°C. The crude brown oil was purified by flash chromatography on silica gel to provide **5c** as a colorless liquid (54%). ^{1}H NMR (400 MHz, $CDCl_3$) δ 7.62 (td, *J* = 7.6, 1.8 Hz, 1H), 7.35 – 7.27 (m, 1H), 7.16 (td, *J* = 7.6, 1.2 Hz, 1H), 7.05 (ddd, *J* = 10.3, 8.2, 1.2 Hz, 1H), 5.76 (dd, *J* = 4.1, 1.8 Hz, 1H), 4.27 (t, *J* = 1.9 Hz, 2H), 3.81 (ddd, *J* = 11.4, 3.0, 1.1 Hz, 1H), 3.45 (ddd, *J* = 11.4, 5.9, 1.8 Hz, 1H), 3.19 – 3.12 (m, 1H), 2.97 (d, *J* = 5.8 Hz, 1H), 2.78 (t, *J* = 4.6 Hz, 1H), 2.61 (dd, *J* = 5.0, 2.7 Hz, 1H); ^{13}C NMR (125 MHz, $CDCl_3$) *δ* 160.1 (d, *J* = 250.0 Hz),130.2(d, *J* = 12.5 Hz),128.2(d, *J* = 2.5 Hz), 127.6(d, *J* = 12.5 Hz), 127.4(d, *J* = 3.75 Hz), 115.6(d, *J* = 21.3 Hz), 85.46, 82.13, 70.4, 59.0 (d, *J* = 5.0 Hz), 58.8 (d, *J* = 37.5 Hz), 50.6, 44.3; HRMS (ESI): calcd for $C_{13}H_{13}FO_3$ $[M+Na]^+$ 259.0741, found 259.0744.

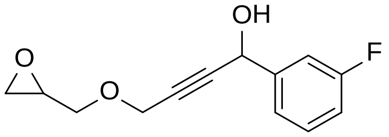

5d

1-(3-fluorophenyl)-4-(oxiran-2-ylmethoxy)but-2-yn-1-ol (5d)

Following procedure A, **1** (484 mg, 2.0 mmol) was allowed to react with 3-fluorobenzaldehyde (774 mg, 6.0 mmol) at -78°C. The crude brown oil was purified by flash chromatography on silica gel to provide **5d** as a colorless liquid (56%). ^{1}H NMR (400 MHz, $CDCl_3$) δ 7.40 – 7.22 (m, 3H), 7.02 (t, *J* = 9.8 Hz, 1H), 5.50 (s, 1H), 4.31 (t, *J* = 1.5 Hz, 2H), 3.85 (dd, *J* = 11.5, 2.9 Hz, 1H), 3.47 (dd, *J* = 11.4, 6.0 Hz, 1H), 3.23 – 3.13 (m, 1H), 2.81 (t, *J* = 4.6 Hz, 1H), 2.63 (dd, *J* = 5.0, 2.7 Hz, 1H), 2.43 (d, *J* = 5.8 Hz, 1H); ^{13}C NMR (125 MHz, $CDCl_3$) *δ* 162.8 (d, *J* = 250.0 Hz), 142.9 (d, *J* = 6.25 Hz), 130.1(d, *J* = 8.75 Hz), 122.1 (d, *J* = 2.5 Hz), 115.1 (d, *J* = 21.2 Hz), 113.2(d, *J* = 2.5 Hz), 86.1, 82.4, 70.5, 63.7, 58.7, 50.6, 44.2; HRMS (ESI): calcd for $C_{13}H_{13}FO_3$ $[M+Na]^+$ 259.0741; found259.0740.

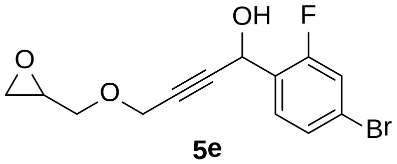

5e

1-(4-bromo-2-fluorophenyl)-4-(oxiran-2-ylmethoxy)but-2-yn-1-ol (5e)

Following procedure A, **1** (484 mg, 2.0 mmol) was allowed to react with 4-bromo-2-fluorobenzaldehyde (1.22 mg, 6.0 mmol) at -78°C. The crude brown oil was purified by flash chromatography on silica gel to provide **5e** as a colorless liquid (66%). ^{1}H NMR (400 MHz, $CDCl_3$) δ 7.51 (t, *J* = 8.1 Hz, 1H), 7.33 (dd, *J* = 8.3, 1.9 Hz, 1H), 7.26 (dd, *J* = 8.3, 1.9 Hz, 1H), 5.72 (d, *J* = 4.9 Hz, 1H), 4.28 (t, *J* = 1.5 Hz, 2H), 3.84 (ddd, *J* = 11.5, 2.9, 1.2 Hz, 1H), 3.45 (ddd, *J* = 11.5, 6.0, 2.1 Hz, 1H), 3.22 – 3.13 (m, 1H), 2.81 (t, *J* = 4.6 Hz, 1H), 2.63 (ddd, *J* = 4.9, 2.7, 1.1 Hz, 1H), 2.55 (s, 1H); ^{13}C NMR (125 MHz, $CDCl_3$) *δ* 159.8 (d, *J* = 250.0 Hz), 129.4 (d, *J* = 3.75 Hz), 127.7, 127.7 (d, *J* = 3.75 Hz), 126.9 (d, *J* = 1.25 Hz), 122.6 (d, *J* = 1.25 Hz), 119.3 (d, *J* = 2.5 Hz), 84.9, 82.5, 70.5, 58.6 (d, *J* = 2.5 Hz), 58.5 (d, *J* = 5.0 Hz), 50.6, 44.3; HRMS (ESI): calcd for $C_{13}H_{12}BrFO_3$ $[M+Na]^+$ 336.9846, found 336.9843.

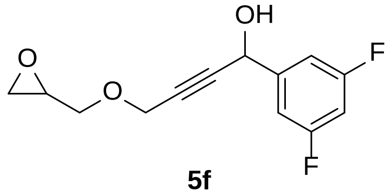

5f

1-(3,5-difluorophenyl)-4-(oxiran-2-ylmethoxy)but-2-yn-1-ol (5f)

Following procedure A, **1** (484 mg, 2.0 mmol) was allowed to react with 3,5-difluorobenzaldehyde (853 mg, 6.0 mmol) at -78°C. The crude brown oil was purified by flash chromatography on silica gel to provide **5f** as a colorless liquid (54%). ^{1}H NMR (500 MHz, $CDCl_3$) δ 7.07 (d, *J* = 6.0 Hz, 2H), 6.76 (tt, *J* = 8.9, 2.4 Hz, 1H), 5.48 (d, *J* = 3.7 Hz, 1H), 4.30 (d, *J* = 1.7 Hz, 2H), 3.86 (dd, *J* = 11.5, 2.7 Hz, 1H), 3.44 (dd, *J* = 11.5, 6.1 Hz, 1H), 3.23 – 3.13 (m, 1H), 2.82 (t, *J* = 4.6 Hz, 1H), 2.63 (dd, *J* = 5.1, 2.8 Hz, 2H); ^{13}C NMR (125 MHz, $CDCl_3$) δ162.97 (dd,*J* = 246, 12.5 Hz), 144.28(t, *J* = 8.75 Hz), 109.40 (dd, *J* = 20, 6.25 Hz),103.52(t,*J* = 25 Hz),85.5, 82.7, 70.58 (d, *J* = 1.25 Hz), 63.3, 58.6, 50.6, 44.2.HRMS (ESI): calcd for$C_{13}H_{12}F_2O_3$$[M+Na]^+$277.0647; found 277.0648.

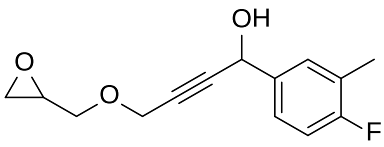

5g

1-(4-fluoro-3-methylphenyl)-4-(oxiran-2-ylmethoxy)but-2-yn-1-ol (5g)

Following procedure B, **1** (484mg, 2.0 mmol) was allowed to react with 4-fluoro-3-methylbenzaldehyde (829 mg, 6.0 mmol) at -78°C. The crude brown oil was purified by flash chromatography on silica gel to provide **5g** as a colorless liquid (69%). ^{1}H NMR (500 MHz, $CDCl_3$) δ 7.34 (dd, *J* = 7.4, 2.3 Hz, 1H), 7.32 – 7.28 (m, 1H), 6.99 (t, *J* = 8.9 Hz, 1H), 5.45 (s, 1H), 4.35 – 4.26 (m, 2H), 3.84 (dd, *J* = 11.4, 2.9 Hz, 1H), 3.46 (dd, *J* = 11.4, 6.0 Hz, 1H), 3.22 – 3.13 (m, 1H), 2.81 (t, *J* = 4.6 Hz, 1H), 2.63 (dd, *J* = 5.0, 2.7 Hz, 1H), 2.46 (s, 1H), 2.29 (s, 3H); ^{13}C NMR (125 MHz, $CDCl_3$) δ161.2 (d, *J* = 245 Hz), 135.9 (d, *J* = 3.75 Hz), 129.8 (d, *J* = 5 Hz), 125.59 (d, *J* = 8.75 Hz), 125.1 (d,*J* = 17.5 Hz), 115.0 (d, *J* = 22.5 Hz), 86.5, 82.3. 70.5, 63.9, 58.7, 50.6, 44.3, 14.5 (d, *J* = 35.0 Hz); HRMS (ESI): calcd for $C_{14}H_{15}FO_3$ $[M+Na]^+$ 273.0897; found 273.0899.

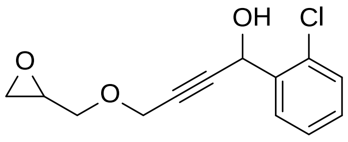

5h

1-(2-chlorophenyl)-4-(oxiran-2-ylmethoxy)but-2-yn-1-ol (5h)

Following procedure B, **1** (484 mg, 2.0 mmol) was allowed to react with 2-chlorobenzaldehyde (840 mg, 6.0 mmol) at -78°C. The crude brown oil was purified by flash chromatography on silica gel to provide **5h** as a colorless liquid (59%). ^{1}H NMR (400 MHz, $CDCl_3$) δ 7.73 (dd, *J* = 7.5, 1.9 Hz, 1H), 7.38 (dd, *J* = 7.6, 1.6 Hz, 1H), 7.34 – 7.24 (m, 2H), 5.86 (d, *J* = 5.5 Hz, 1H), 4.29 (t, *J* = 1.9 Hz, 2H), 3.83 (dt, *J* = 11.4, 2.9 Hz, 1H), 3.47 (ddd, *J* = 11.4, 5.9, 4.0 Hz, 1H), 3.23 – 3.12 (m, 1H), 2.85 – 2.73 (m, 2H), 2.62 (ddd, *J* = 4.7, 2.7, 1.4 Hz, 1H); ^{13}C NMR (125 MHz, $CDCl_3$) δ 137.8, 132.5, 129.6, 129.5, 128.1, 127.2, 85.6, 81.9, 70.3, 61.6, 58.7, 50.5, 44.3;HRMS (ESI): calcd for $C_{13}H_{13}ClO_3$ $[M+Na]^+$ 275.0445; found 275.0446.

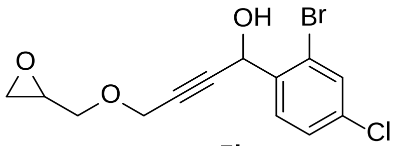

5i

1-(2-bromo-4-chlorophenyl)-4-(oxiran-2-ylmethoxy)but-2-yn-1-ol (5i)

Following procedure B, **1** (484 mg, 2.0 mmol) was allowed to react with 2-bromo-4-chlorobenzaldehyde (1.32 g, 6.0 mmol) at -78°C. The crude brown oil was purified by flash chromatography on silica gel to provide **5i** as a colorless liquid (58%). ^{1}H NMR (400 MHz, $CDCl_3$) δ 7.72 (d, *J* = 2.5 Hz, 1H), 7.47 (d, *J* = 8.5 Hz, 1H), 7.16 (dd, *J* = 8.5, 2.6 Hz, 1H), 5.74 (d, *J* = 5.1 Hz, 1H), 4.34 – 4.23 (m, 2H), 3.84 (dt, *J* = 11.5, 3.2 Hz, 1H), 3.45 (ddd, *J* = 11.4, 5.9, 4.1 Hz, 1H), 3.21 – 3.13 (m, 1H), 3.00 (dd, *J* = 5.3, 2.4 Hz, 1H), 2.81 (dd, *J* = 4.9, 4.2 Hz, 1H), 2.63 (ddd, *J* = 4.7, 2.7, 1.3 Hz, 1H); ^{13}C NMR (125 MHz, $CDCl_3$) *δ* 141.1, 134.0, 129.8, 128.4, 120.1, 85.0, 82.5, 70.5, 63.5, 58.7, 50.6, 44.3; HRMS (ESI): calcd for $C_{13}H_{12}BrClO_3$ $[M+Na]^+$ 352.9551; found 352.9553.

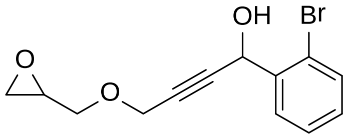

5j

1-(2-bromophenyl)-4-(oxiran-2-ylmethoxy)but-2-yn-1-ol (5j)

Following procedure B, **1** (484 mg, 2.0 mmol) was allowed to react with 2-bromobenzaldehyde (1.10 g, 6.0 mmol) at -78°C. The crude brown oil was purified by flash chromatography on silica gel to provide **5j** as a colorless liquid (44%). ^{1}H NMR (400 MHz, $CDCl_3$) δ 7.75 (dd, *J* = 7.8, 1.7 Hz, 1H), 7.57 (dd, *J* = 8.0, 1.2 Hz, 1H), 7.37 (td, *J* = 7.6, 1.2 Hz, 1H), 7.20 (td, *J* = 7.7, 1.7 Hz, 1H), 5.84 (d, *J* = 4.9 Hz, 1H), 4.30 (t, *J* = 1.9 Hz, 2H), 3.84 (dt, *J* = 11.4, 3.1 Hz, 1H), 3.48 (ddd, *J* = 11.4, 5.9, 4.5 Hz, 1H), 3.21 – 3.12 (m, 1H), 2.81 (t, *J* = 4.4 Hz, 1H), 2.63 (ddd, *J* = 4.8, 2.6, 1.6 Hz, 1H), 2.57 (d, *J* = 5.4 Hz, 1H); ^{13}C NMR (125 MHz, $CDCl_3$) δ 139.3, 133.0, 129.9, 128.4, 127.9, 122.5, 85.5, 82.3, 70.4, 64.0, 58.7, 50.6, 44.3. HRMS (ESI): calcd for $C_{13}H_{13}BrO_3[M+Na]^+$ 318.9940; found 318.9941.

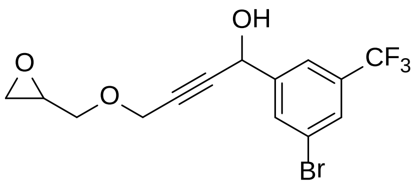

5k

1-(3-bromo-5-(trifluoromethyl)phenyl)-4-(oxiran-2-ylmethoxy)but-2-yn-1-ol (5k)

Following procedure B, **1** (484 mg, 2.0 mmol) was allowed to react with 3-bromo-5-(trifluoromethyl)benzaldehyde (1.52 mg, 6.0 mmol) at -78°C. The crude brown oil was purified by flash chromatography on silica gel to provide **5k** as a colorless liquid (45%). ^{1}H NMR (400 MHz, $CDCl_3$) δ 7.87 (s, 1H), 7.73 (s, 2H), 5.54 (d, *J* = 5.5 Hz, 1H), 4.31 (d, *J* = 1.0 Hz, 2H), 3.88 (dd, *J* = 11.5, 2.7 Hz, 1H), 3.44 (dd, *J* = 11.5, 6.1 Hz, 1H), 3.23 – 3.14 (m, 1H), 2.83 (t, *J* = 4.6 Hz, 1H), 2.75 (dd, *J* = 5.8, 3.4 Hz, 1H), 2.64 (dd, *J* = 4.9, 2.7 Hz, 1H); ^{13}C NMR (126 MHz, $CDCl_3$) δ 143.5, 133.0, 132.52 (q, *J* = 32.5 Hz), 128.2 (q, *J* = 3.75 Hz), 122.8, 122.1 (q, *J* = 3.75 Hz), 123.0 (q, *J* = 271 Hz), 85.3, 83.3, 70.7, 63.1, 58.6, 50.6, 44.2. HRMS (ESI): calcd for $C_{14}H_{12}BrF_3O_3$ $[M+Na]^+$ 386.9814; found 386.9816.

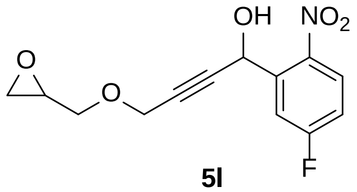

5l

1-(5-fluoro-2-nitrophenyl)-4-(oxiran-2-ylmethoxy)but-2-yn-1-ol (5l)

Following procedure B, **1** (484 mg, 2.0 mmol) was allowed to react with 5-fluoro-2-nitrobenzaldehyde (1.01 g, 6.0 mmol) at -78°C. The crude brown oil was purified by flash chromatography on silica gel to provide **5l** as a colorless liquid (50%). ^{1}H NMR (400 MHz, $CDCl_3$) δ 8.09 (dd, *J* = 9.0, 5.0 Hz, 1H), 7.68 (dd, *J* = 9.3, 2.8 Hz, 1H), 7.17 (ddd, *J* = 9.4, 7.0, 2.8 Hz, 1H), 6.13 (s, 1H), 4.26 (d, *J* = 1.7 Hz, 2H), 3.84 (ddd, *J* = 11.5, 4.8, 2.8 Hz, 1H), 3.42 (ddd, *J* = 11.5, 7.3, 6.0 Hz, 1H), 3.25 (s, 1H), 3.21 – 3.12 (m, 1H), 2.82 (t, *J* = 4.6 Hz, 1H), 2.64 (dt, *J* = 5.0, 2.9 Hz, 1H); ^{13}C NMR (126 MHz, $CDCl_3$) δ 165.3 (d, *J* = 256.2 Hz), 143. 6, 139.3 (d, *J* = 8.8 Hz), 128.1 (d, *J* = 8.8 Hz), 116.2 (d, *J* = 16.3 Hz), 116.0 (d, *J* = 13.6 Hz), 84.0, 82.6, 70.6,

60.8, 58.6, 50.6, 44.3; HRMS (ESI): calcd for $C_{13}H_{12}FNO_5$ $[M+Na]^+$ 304.0592; found 304.0592.

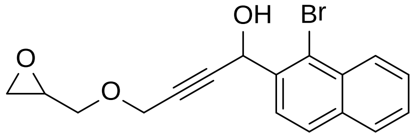

5m

1-(1-bromonaphthalen-2-yl)-4-(oxiran-2-ylmethoxy)but-2-yn-1-ol (5m)

Following procedure B, **1** (484 mg, 2.0 mmol) was allowed to react with 1-bromo-2-naphthaldehyde (1.41 g, 6.0 mmol) at -78°C. The crude brown oil was purified by flash chromatography on silica gel to provide **5m** as a colorless liquid (56%). ^{1}H NMR (400 MHz, $CDCl_3$) δ 8.34 (d, *J* = 8.7 Hz, 1H), 7.91 – 7.79 (m, 3H), 7.62 (ddd, *J* = 8.4, 6.9, 1.4 Hz, 1H), 7.55 (ddd, *J* = 8.1, 6.9, 1.3 Hz, 1H), 6.19 (d, *J* = 4.8 Hz, 1H), 4.30 (t, *J* = 1.9 Hz, 2H), 3.83 (dt, *J* = 11.4, 3.3 Hz, 1H), 3.47 (ddd, *J* = 11.4, 5.9, 4.8 Hz, 1H), 3.20 – 3.12 (m, 1H), 2.79 (ddd, *J* = 5.2, 4.2, 1.2 Hz, 1H), 2.71 (dd, *J* = 4.9, 1.3 Hz, 1H), 2.62 (dt, *J* = 4.9, 2.4 Hz, 1H); ^{13}C NMR (125 MHz, $CDCl_3$) δ 137.4, 134.4, 132.2, 128.4, 128.2, 127.7, 127.6, 127.0, 124.9, 122.7, 85.9, 82.4, 70.4, 64.9, 58.8, 50.6, 44.3. HRMS (ESI): calcd for $C_{17}H_{15}BrO_3[M+Na]^+$ 369.0097; found 369.0094.

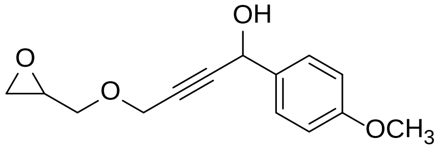

5n

1-(4-methoxyphenyl)-4-(oxiran-2-ylmethoxy)but-2-yn-1-ol (5n)

Following procedure B, **1** (484 mg, 2.0 mmol) was allowed to react with 4-methoxybenzaldehyde (817 mg, 6.0 mmol) at -78°C. The crude brown oil was purified by flash chromatography on silica gel to provide **5n** as a colorless liquid (55%). ^{1}H NMR (400 MHz, $CDCl_3$) δ 7.45 (d, *J* = 8.7 Hz, 2H), 6.91 (d, *J* = 8.7 Hz, 2H), 5.46 (d, *J* = 5.9 Hz, 1H), 4.31 (t, *J* = 2.1 Hz, 2H), 3.86 – 3.82 (m, 1H), 3.82 (s, 3H), 3.48 (dd, *J* = 11.4, 5.9 Hz, 1H), 3.21 – 3.13 (m, 1H), 2.81 (dd, *J* = 5.0, 4.1 Hz, 1H), 2.63 (dd, *J* = 5.0, 2.7 Hz, 1H), 2.23 (d, *J* = 5.9 Hz, 1H); ^{13}C NMR (125 MHz,

$CDCl_3$) δ 159.4, 133.1, 127.8, 113.7, 87.1, 81.3, 70.2, 63.6, 58.6, 55.0, 50.3, 44.0; HRMS (ESI): calcd for $C_{14}H_{16}O_4[M+Na]^+$ 271.0941; found 271.0943.

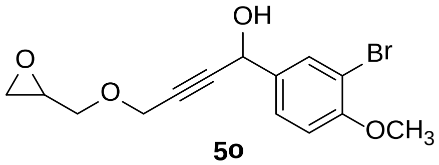

5o

1-(3-bromo-4-methoxyphenyl)-4-(oxiran-2-ylmethoxy)but-2-yn-1-ol (5o)

Following procedure B, **1** (484 mg, 2.0 mmol) was allowed to react with 3-bromo-4-methoxybenzaldehyde (1.29 mg, 6.0 mmol) at -78°C. The crude brown oil was purified by flash chromatography on silica gel to provide **5o** as a colorless liquid (52%). ^{1}H NMR (400 MHz, $CDCl_3$) δ 7.72 (d, *J* = 2.2 Hz, 1H), 7.43 (dd, *J* = 8.5, 2.2 Hz, 1H), 6.89 (d, *J* = 8.5 Hz, 1H), 5.44 (s, 1H), 4.31 (d, *J* = 1.6 Hz, 2H), 3.90 (s, 3H), 3.85 (dd, *J* = 11.4, 2.9 Hz, 1H), 3.47 (ddd, *J* = 11.5, 6.0, 0.8 Hz, 1H), 3.23 – 3.14 (m, 1H), 2.82 (t, *J* = 4.6 Hz, 1H), 2.64 (dd, *J* = 5.0, 2.7 Hz, 1H), 2.36 (d, *J* = 5.8 Hz, 1H); ^{13}C NMR (101 MHz, $CDCl_3$) δ 156.3, 134.4, 131.9, 126.9, 112.2, 112.1, 86.3, 82.8, 70.7, 63.7, 58.8, 56.5, 50.5, 44.3; HRMS (ESI): calcd for$C_{14}H_{15}BrO_4$ $[M+Na]^+$ 349.0046; found 349.0047.

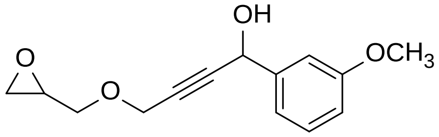

5p

1-(3-methoxyphenyl)-4-(oxiran-2-ylmethoxy)but-2-yn-1-ol (5p)

Following procedure B, **1** (484 mg, 2.0 mmol) was allowed to react with 3-methoxybenzaldehyde (817 mg, 6.0 mmol) at -78°C. The crude brown oil was purified by flash chromatography on silica gel to provide **5p** as a colorless liquid (50%). ^{1}H NMR (400 MHz, $CDCl_3$) δ 7.30 (t, *J* = 7.9 Hz, 1H), 7.15 – 7.07 (m, 2H), 6.87 (ddd, *J* = 8.3, 2.6, 1.0 Hz, 1H), 5.48 (d, *J* = 5.7 Hz, 1H), 4.30 (t, *J* = 1.9 Hz, 2H), 3.86 – 3.82 (m, 1H), 3.83 (s, 3H), 3.48 (ddd, *J* = 11.4, 5.9, 0.8 Hz, 1H), 3.23 – 3.13 (m, 1H), 2.80 (t, *J* = 4.6 Hz, 1H), 2.63 (dd, *J* = 5.0, 2.7 Hz, 1H), 2.36 (s, 1H); ^{13}C NMR (100 MHz, $CDCl_3$) δ 160.2, 142.2, 129.7, 118.9, 114.3, 112.4, 86.7, 82.5, 70.6,

64.7, 58.9, 55.4, 50.5, 44.3; HRMS (ESI): calcd for $C_{14}H_{16}O_4$ $[M+Na]^+$ 271.0941; found 271.0943.

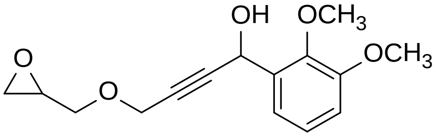

5q

1-(2,3-dimethoxyphenyl)-4-(oxiran-2-ylmethoxy)but-2-yn-1-ol (5q)

Following procedure B, **1** (484 mg, 2.0 mmol) was allowed to react with 4-fluoro-3-methylbenzaldehyde (997 mg, 6.0 mmol) at -78°C. The crude brown oil was purified by flash chromatography on silica gel to provide **5q** as a colorless liquid (55%). ^{1}H NMR (400 MHz, $CDCl_3$) δ 7.12 – 7.02 (m, 2H), 6.91 (dd, *J* = 7.3, 2.4 Hz, 1H), 5.66 (d, *J* = 7.1 Hz, 1H), 4.29 (dd, *J* = 3.5, 1.8 Hz, 2H), 3.95 (s, 3H), 3.87 (s, 3H), 3.81 (dd, *J* = 11.4, 3.0 Hz, 1H), 3.47 (ddd, *J* = 11.3, 5.8, 1.2 Hz, 1H), 3.20 – 3.12 (m, 1H), 3.09 (dd, *J* = 7.1, 2.2 Hz, 1H), 2.79 (ddd, *J* = 5.0, 4.2, 0.8 Hz, 1H), 2.62 (dd, *J* = 5.0, 2.7 Hz, 1H); ^{13}C NMR (125 MHz, $CDCl_3$) δ 152.6, 146.5, 134.3, 124.2, 119.4, 112.9, 86.9, 81.4, 70.3, 61.1, 61.1, 58.8, 55.8, 50.5, 44.3. HRMS (ESI): calcd for $C_{15}H_{18}O_5[M+Na]^+$ 301.1052; found 301.1053.

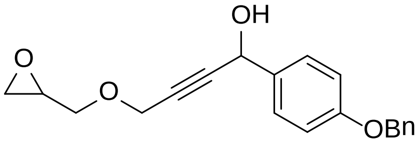

5r

1-(4-(benzyloxy)phenyl)-4-(oxiran-2-ylmethoxy)but-2-yn-1-ol (5r)

Following procedure B, **1** (484 mg, 2.0 mmol) was allowed to react with 4-(benzyloxy)benzaldehyde (1.27 mg, 6.0 mmol) at -78°C. The crude brown oil was purified by flash chromatography on silica gel to provide **5r** as a colorless liquid (48%). ^{1}H NMR (400 MHz, $CDCl_3$) δ 7.49 – 7.29 (m, 7H), 6.98 (d, *J* = 8.7 Hz, 2H), 5.46 (s, 1H), 5.08 (s, 2H), 4.30 (t, *J* = 2.0 Hz, 2H), 3.83 (dd, *J* = 11.4, 3.0 Hz, 1H), 3.48 (dd, *J* = 11.4, 5.9 Hz, 1H), 3.22 – 3.12 (m, 1H), 2.80 (t, *J* = 4.6 Hz, 1H), 2.63 (dd, *J* = 5.0, 2.7 Hz, 1H), 2.38 (s, 1H); ^{13}C NMR (125 MHz, $CDCl_3$) *δ* 158.9, 136.8, 132.9,

128.6, 128.0, 128.0, 127.40, 115.0, 86.7, 82.1, 70. 5, 70.1, 64.2, 58.8, 50.6, 44.3; HRMS (ESI): calcd for $C_{20}H_{20}O_4$ $[M+Na]^+$ 347.1254; found 347.1251.

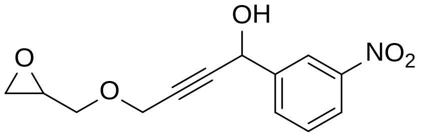

5s

1-(3-nitrophenyl)-4-(oxiran-2-ylmethoxy)but-2-yn-1-ol (5s)

Following procedure B, **1** (484 mg, 2.0 mmol) was allowed to react with 3-nitrobenzaldehyde (907 mg, 6.0 mmol) at -78°C. The crude brown oil was purified by flash chromatography on silica gel to provide **5s** as a colorless liquid (44%). ^{1}H NMR (400 MHz, $CDCl_3$) δ 8.42 (t, *J* = 1.9 Hz, 1H), 8.20 (dd, *J* = 8.2, 1.4 Hz, 1H), 7.88 (d, *J* = 7.7 Hz, 1H), 7.57 (t, *J* = 8.0 Hz, 1H), 5.62 (d, *J* = 5.6 Hz, 1H), 4.32 (d, *J* = 1.7 Hz, 2H), 3.89 (dd, *J* = 11.5, 2.7 Hz, 1H), 3.46 (dd, *J* = 11.5, 6.1 Hz, 1H), 3.24 – 3.15 (m, 1H), 2.83 (t, *J* = 4.6 Hz, 1H), 2.70 – 2.60 (m, 2H); ^{13}C NMR (100 MHz, $CDCl_3$) *δ* 148.6, 142.8, 132.4, 129.5, 123.1, 121.5, 85.6, 83.5, 70.7, 63.5, 58.7, 50.6, 44.2; HRMS (ESI): calcd for $C_{13}H_{13}NO_5$ $[M+Na]^+$ 286.0686; found 286.0688.

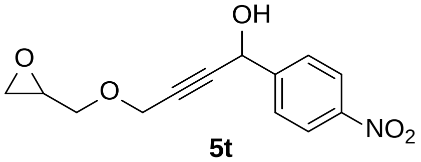

5t

1-(4-nitrophenyl)-4-(oxiran-2-ylmethoxy)but-2-yn-1-ol (5t)

Following procedure B, **1** (484 mg, 2.0 mmol) was allowed to react with 4-nitrobenzaldehyde (907 mg, 6.0 mmol) at -78°C. The crude brown oil was purified by flash chromatography on silica gel to provide **5t** as a colorless liquid (54%). ^{1}H NMR (400 MHz, $CDCl_3$) δ 8.26 (d, *J* = 8.8 Hz, 2H), 7.73 (d, *J* = 8.2 Hz, 2H), 5.63 (d, *J* = 5.7 Hz, 1H), 4.33 (d, *J* = 1.8 Hz, 2H), 3.89 (dd, *J* = 11.5, 2.7 Hz, 1H), 3.45 (dd, *J* = 11.5, 6.1 Hz, 1H), 3.24 – 3.16 (m, 1H), 2.84 (ddd, *J* = 4.9, 4.2, 0.6 Hz, 1H), 2.72 (dd, *J* = 5.7, 1.8 Hz, 1H), 2.65 (dd, *J* = 4.9, 2.7 Hz, 1H); ^{13}C NMR (100 MHz, $CDCl_3$) *δ* 148.2, 147.2, 127.3, 123.8, 85.5, 83.8, 70.9, 63.7, 58.8, 50.6, 44.2; HRMS (ESI): calcd for $C_{13}H_{13}NO_5$ $[M+Na]^+$ 286.0686; found 286.0687.

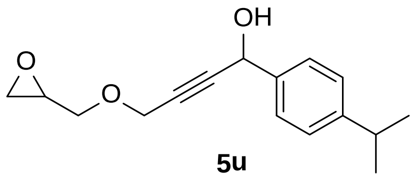

5u

1-(4-isopropylphenyl)-4-(oxiran-2-ylmethoxy)but-2-yn-1-ol (5u)

Following procedure B, **1** (484 mg, 2.0 mmol) was allowed to react with 4-isopropylbenzaldehyde (889 mg, 6.0 mmol) at -78°C. The crude brown oil was purified by flash chromatography on silica gel to provide **5u** as a colorless liquid (44%). ^{1}H NMR (400 MHz, $CDCl_3$) δ 7.45 (d, *J* = 8.2 Hz, 2H), 7.24 (d, *J* = 6.5 Hz, 2H), 5.48 (d, *J* = 6.0 Hz, 1H), 4.31 (dd, *J* = 2.6, 1.8 Hz, 2H), 3.84 (dd, *J* = 11.4, 3.0 Hz, 1H), 3.49 (dd, *J* = 11.4, 5.9 Hz, 1H), 3.22 – 3.14 (m, 1H), 2.98 – 2.85 (m, 1H), 2.81 (t, *J* = 4.6 Hz, 1H), 2.63 (dd, *J* = 5.0, 2.7 Hz, 1H), 2.22 (d, *J* = 6.1 Hz, 1H), 1.25 (d, *J* = 6.9 Hz, 6H); ^{13}C NMR (100 MHz, $CDCl_3$) *δ* 149.4, 138.2, 126.7, 126.7, 87.0, 82.3, 70.6, 64.7, 58.9, 50.5, 44.3, 33.9, 23.9; HRMS (ESI): calcd for $C_{16}H_{20}O_3$ $[M+Na]^+$ 283.1305; found 283.1305.

2. Copies of ^{1}H and ^{13}C NMR of products

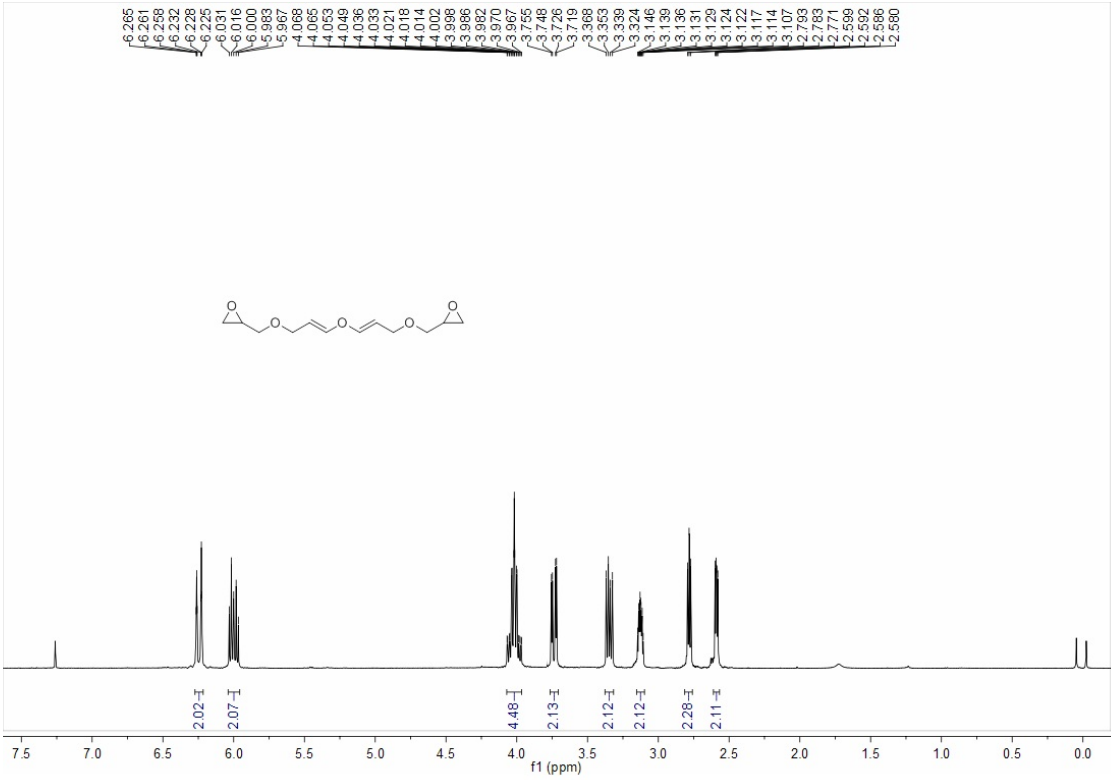

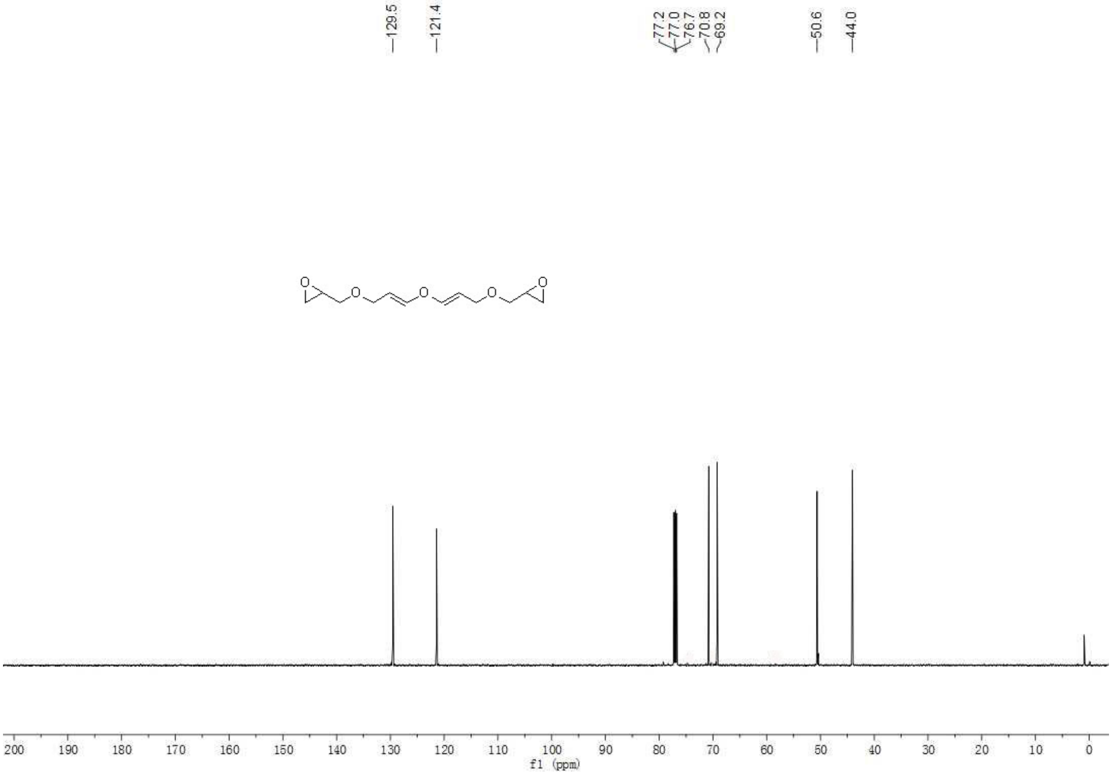

1

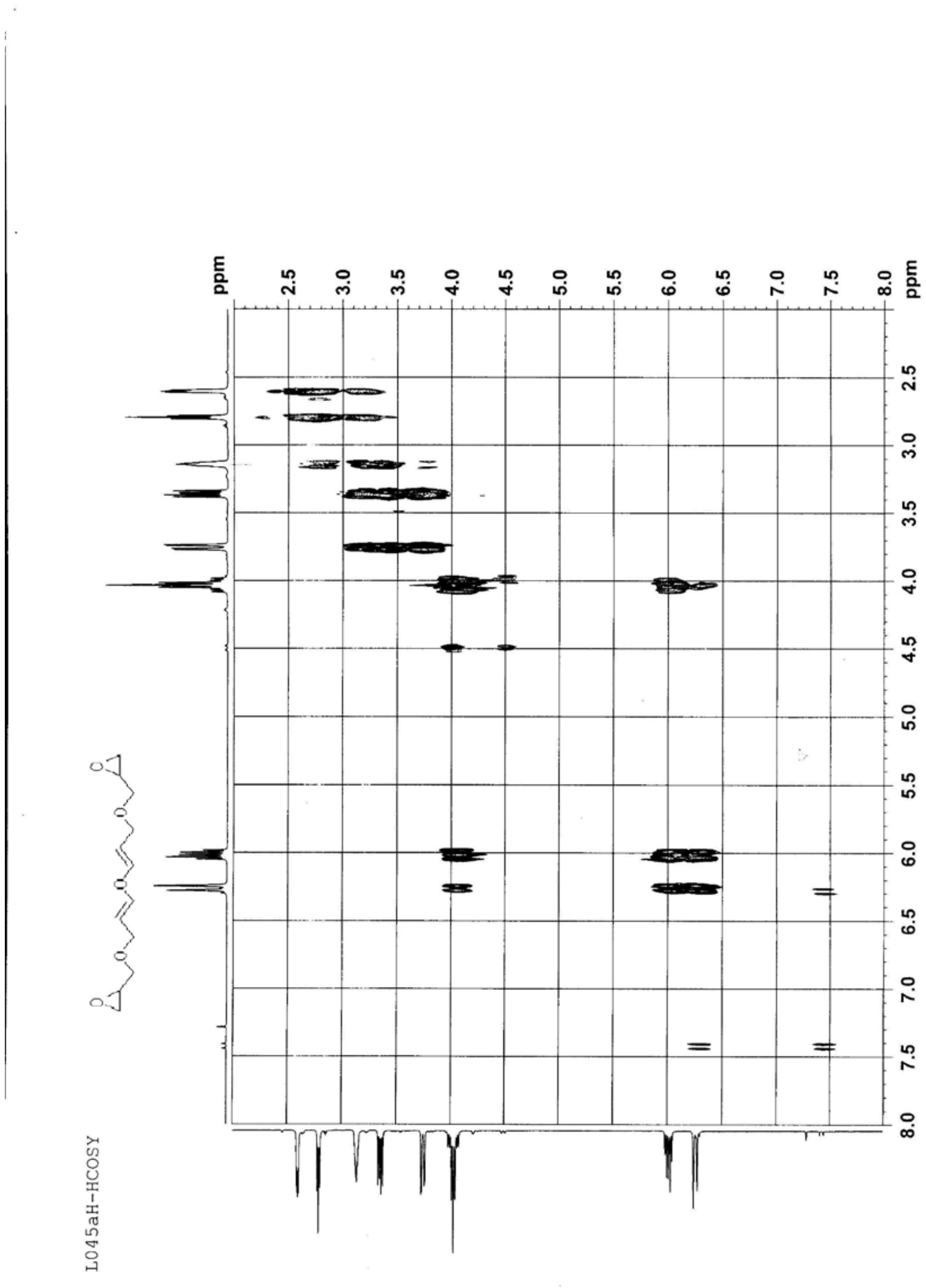

1

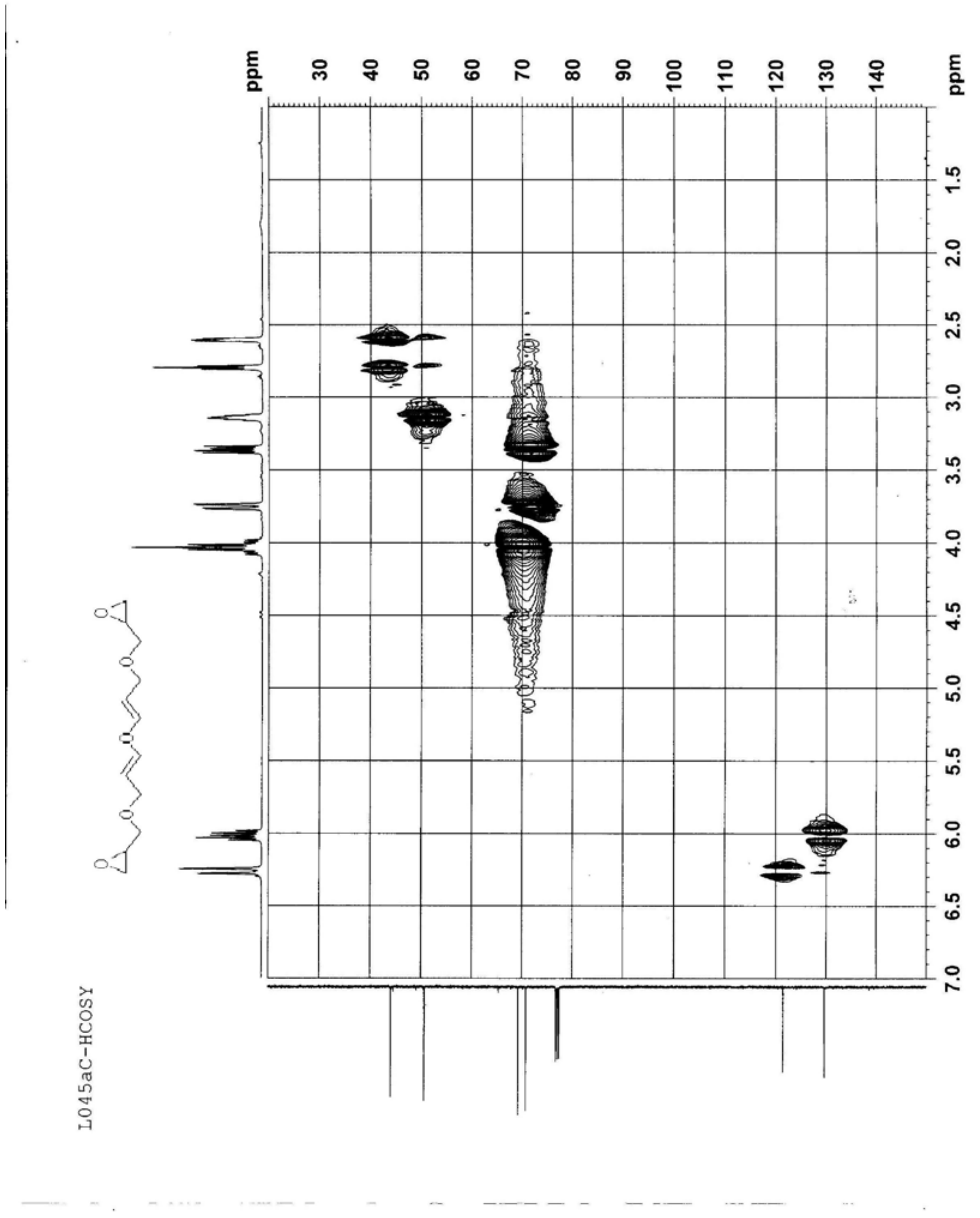
L045aC-HCOSY
ppm
30
40
50
60
70
80
90
100
110
120
130
140
ppm
1.5
2.0
2.5
3.0
3.5
4.0
4.5
5.0
5.5
6.0
6.5
7.0

1

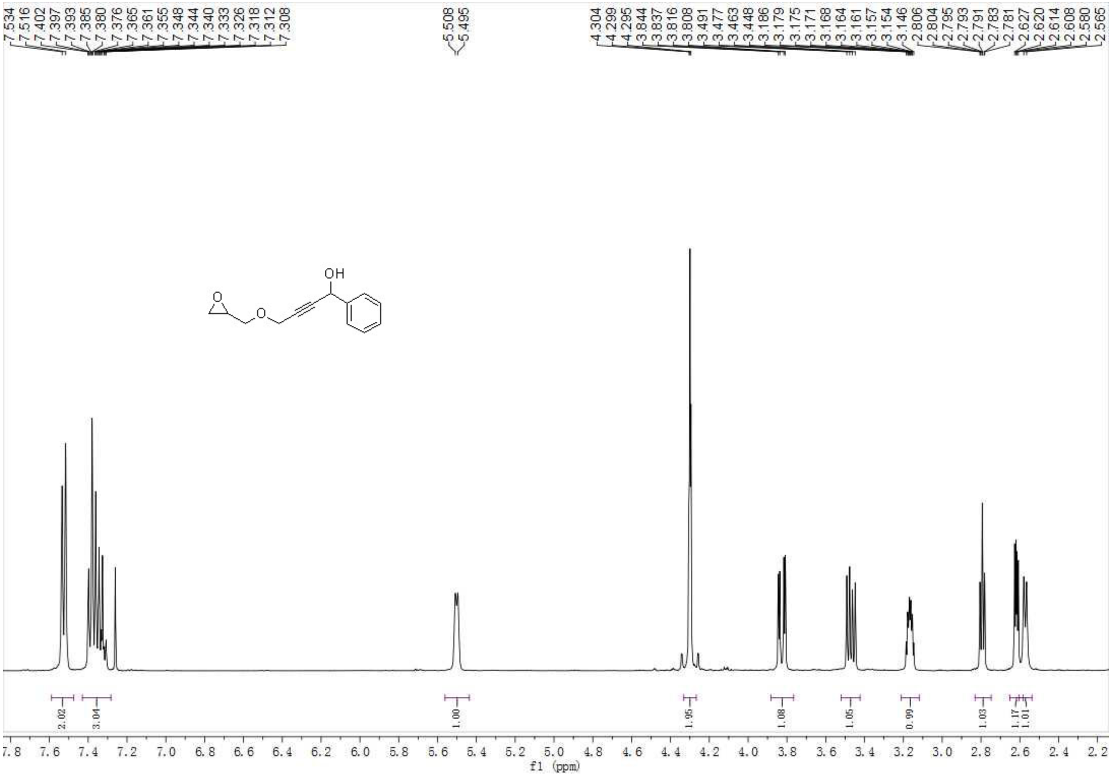

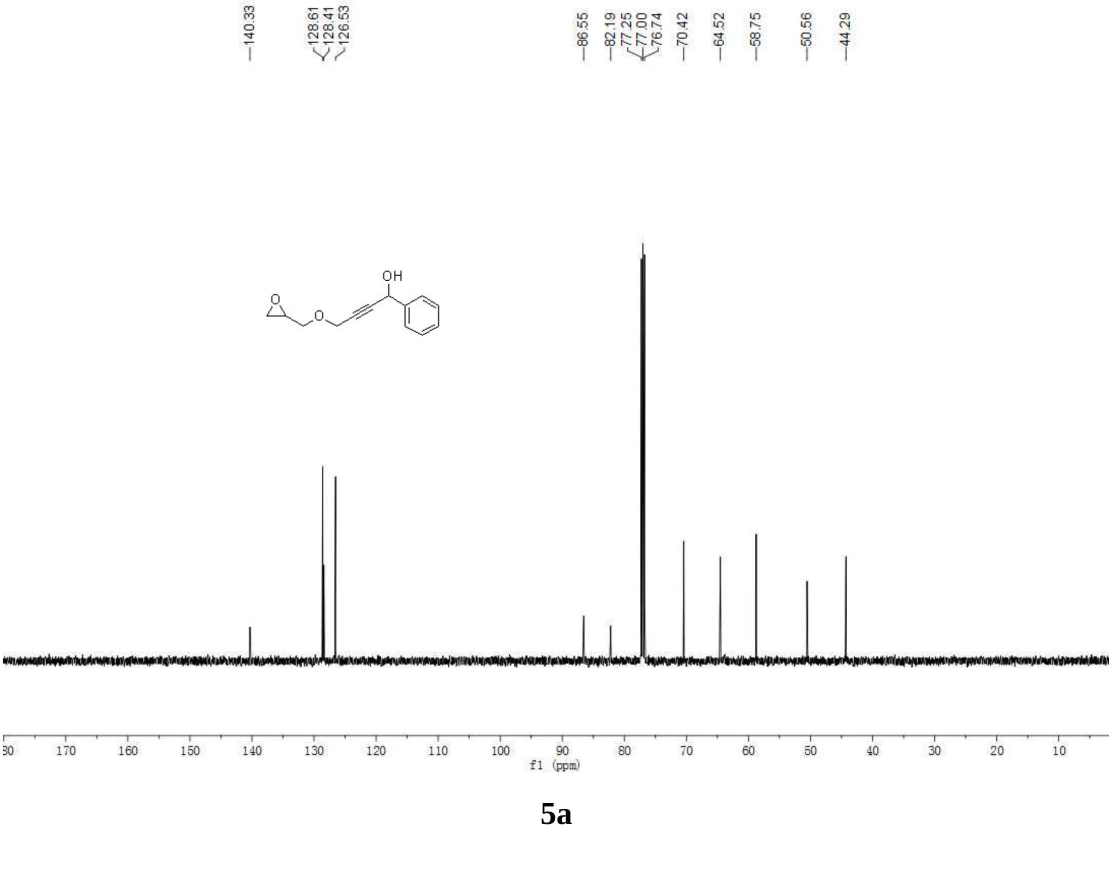

5a

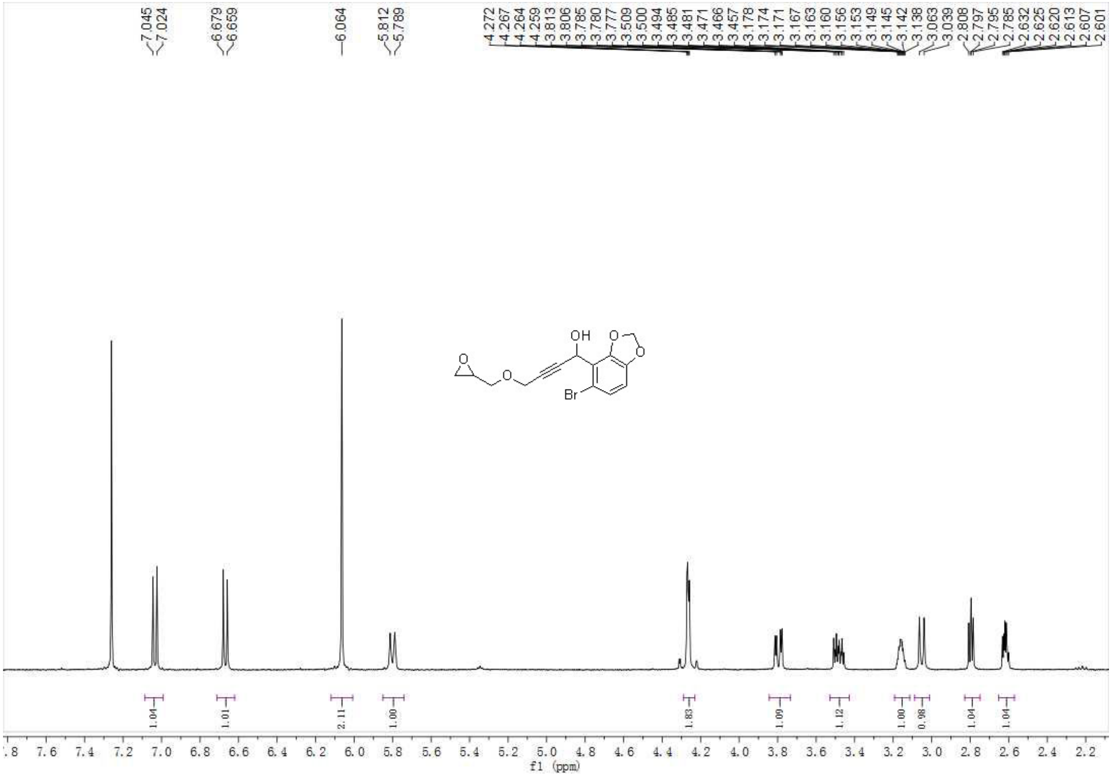

7.045
7.024
6.679
6.659
6.064
5.812
5.789
4.272
4.267
4.264
4.259
3.813
3.806
3.785
3.780
3.777
3.509
3.500
3.494
3.485
3.481
3.471
3.466
3.457
3.178
3.174
3.171
3.167
3.163
3.160
3.156
3.153
3.149
3.145
3.142
3.138
3.063
3.039
2.808
2.797
2.795
2.785
2.632
2.625
2.620
2.613
2.607
2.601
OH
O
O
O
O
Br
1.04
1.01
2.11
1.00
1.83
1.09
1.12
1.00
0.98
1.04
1.04
f1 (ppm)

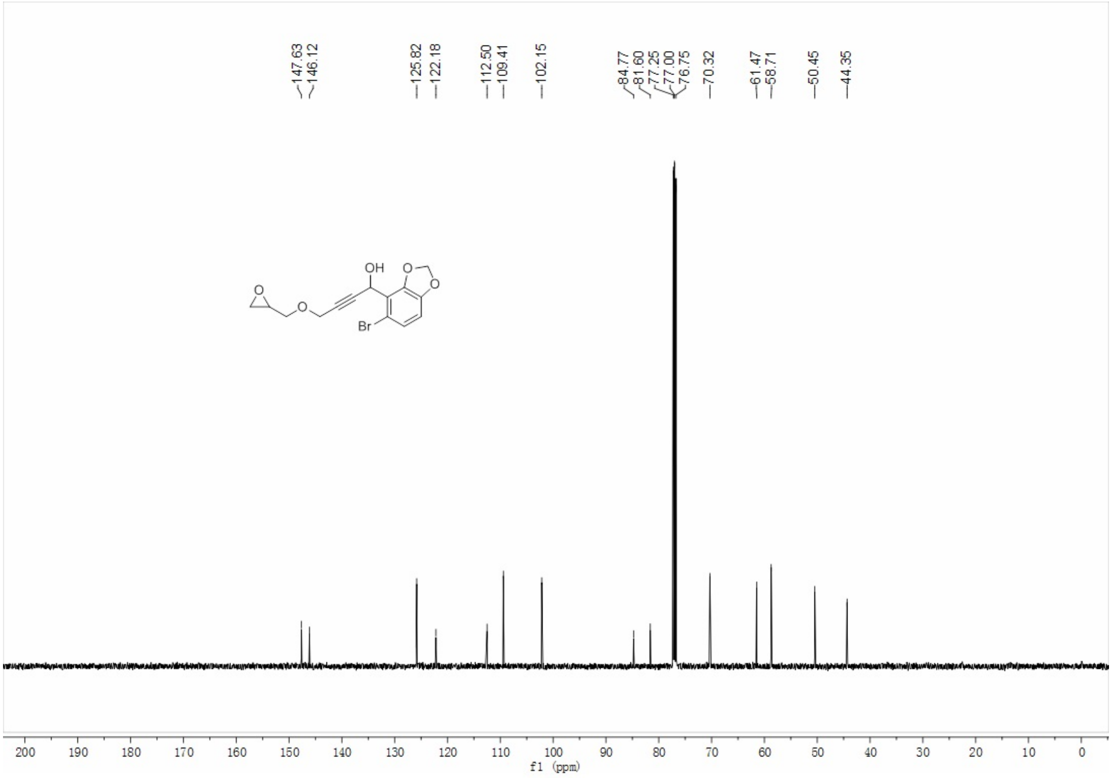

147.63
146.12
125.82
122.18
112.50
109.41
102.15
84.77
81.60
77.25
77.00
76.75
70.32
61.47
58.71
50.45
44.35
OH
O
O
O
O
Br
f1 (ppm)

5b

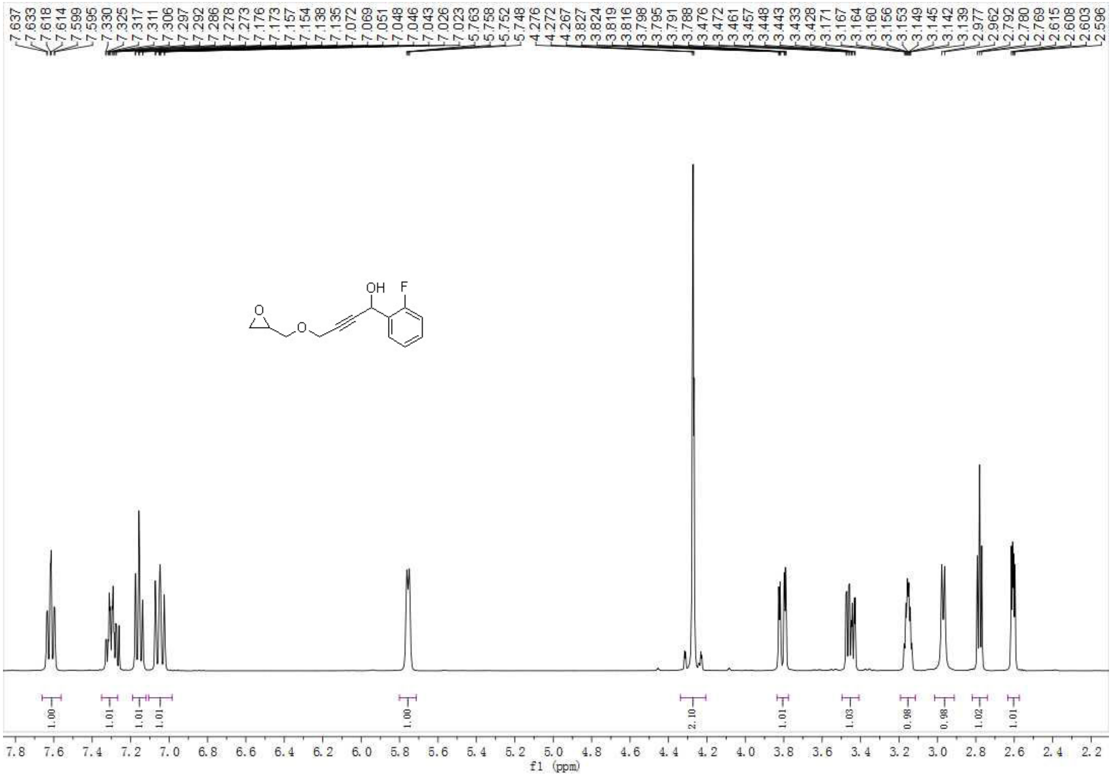
7.637
7.633
7.618
7.614
7.599
7.595
7.330
7.325
7.317
7.311
7.306
7.297
7.292
7.286
7.278
7.273
7.176
7.173
7.157
7.154
7.138
7.135
7.072
7.069
7.051
7.048
7.046
7.043
7.026
7.023
5.763
5.758
5.752
5.748
4.276
4.272
4.267
3.827
3.824
3.819
3.816
3.798
3.795
3.791
3.788
3.476
3.472
3.461
3.457
3.448
3.443
3.433
3.428
3.171
3.167
3.164
3.160
3.156
3.153
3.149
3.145
3.142
3.139
2.977
2.962
2.792
2.780
2.769
2.615
2.608
2.603
2.596
OH
F
O
O
1.00
1.01
1.01
1.01
1.00
2.10
1.01
1.03
0.98
0.98
1.02
1.01
7.8 7.6 7.4 7.2 7.0 6.8 6.6 6.4 6.2 6.0 5.8 5.6 5.4 5.2 5.0 4.8 4.6 4.4 4.2 4.0 3.8 3.6 3.4 3.2 3.0 2.8 2.6 2.4 2.2
f1 (ppm)

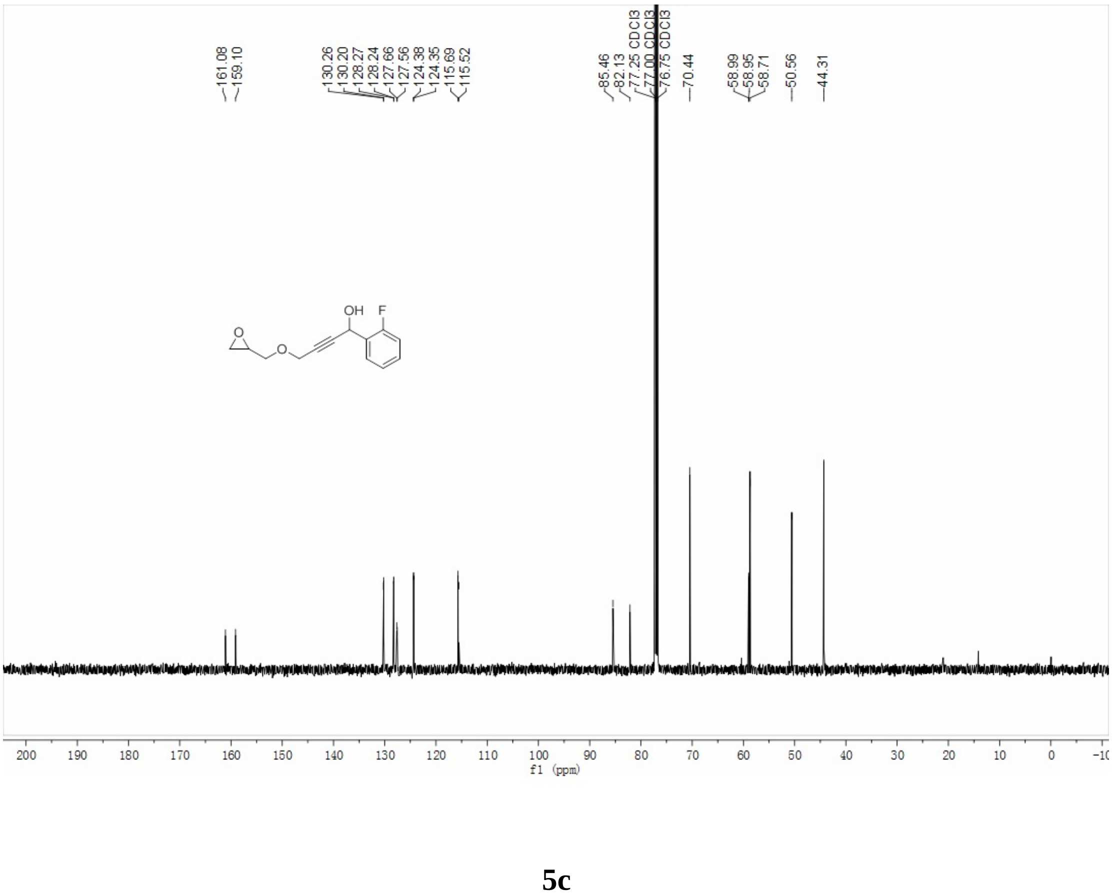
161.08
159.10
130.26
130.20
128.27
128.24
127.66
127.56
124.38
124.35
115.69
115.52
85.46
82.13
77.25 CDCl3
77.00 CDCl3
76.75 CDCl3
70.44
58.99
58.95
58.71
50.56
44.31
OH
F
O
O
200 190 180 170 160 150 140 130 120 110 100 90 80 70 60 50 40 30 20 10 0 -10
f1 (ppm)

5c

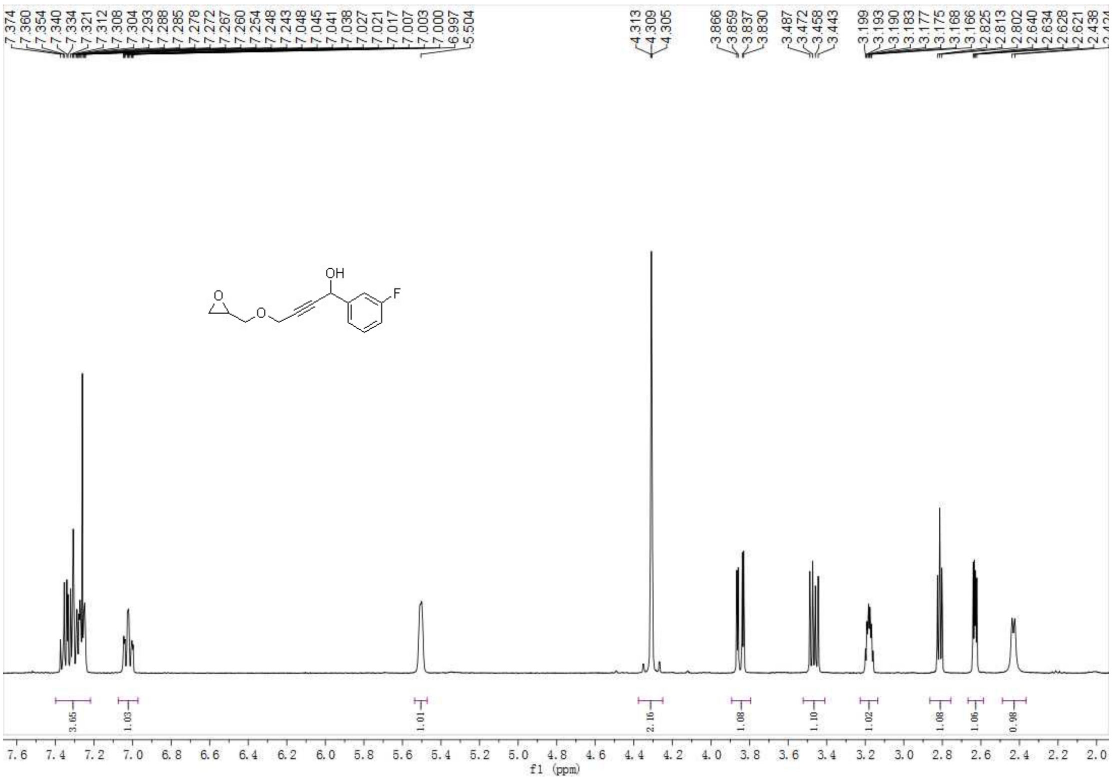

7.374
7.360
7.354
7.340
7.334
7.321
7.312
7.308
7.304
7.293
7.288
7.285
7.278
7.272
7.267
7.260
7.254
7.248
7.243
7.048
7.045
7.041
7.038
7.027
7.021
7.017
7.007
7.003
7.000
6.997
5.504
4.313
4.309
4.305
3.866
3.859
3.837
3.830
3.487
3.472
3.458
3.443
3.199
3.193
3.190
3.183
3.177
3.175
3.168
3.166
2.825
2.813
2.802
2.640
2.634
2.628
2.621
2.438
OH
F
O
O
3.65
1.03
1.01
2.16
1.08
1.10
1.02
1.08
1.06
0.98
f1 (ppm)

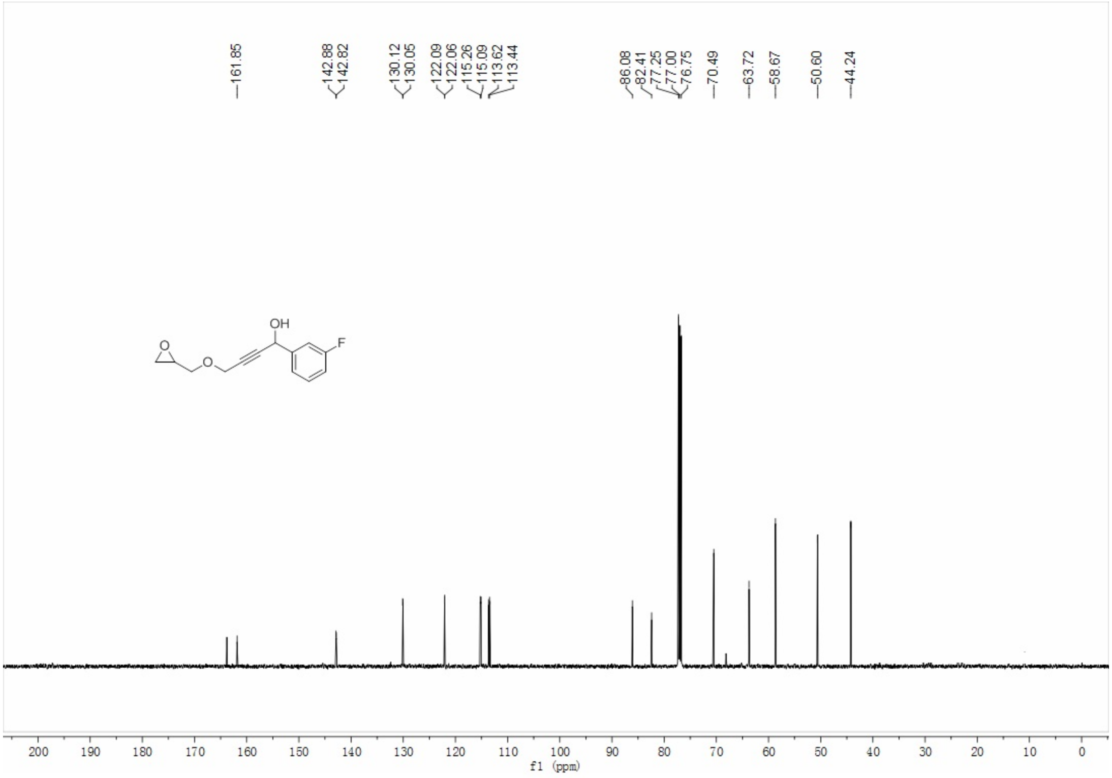

161.85
142.88
142.82
130.12
130.05
122.09
122.06
115.26
115.09
113.62
113.44
86.08
82.41
77.25
77.00
76.75
70.49
63.72
58.67
50.60
44.24
OH
F
O
O
f1 (ppm)

5d

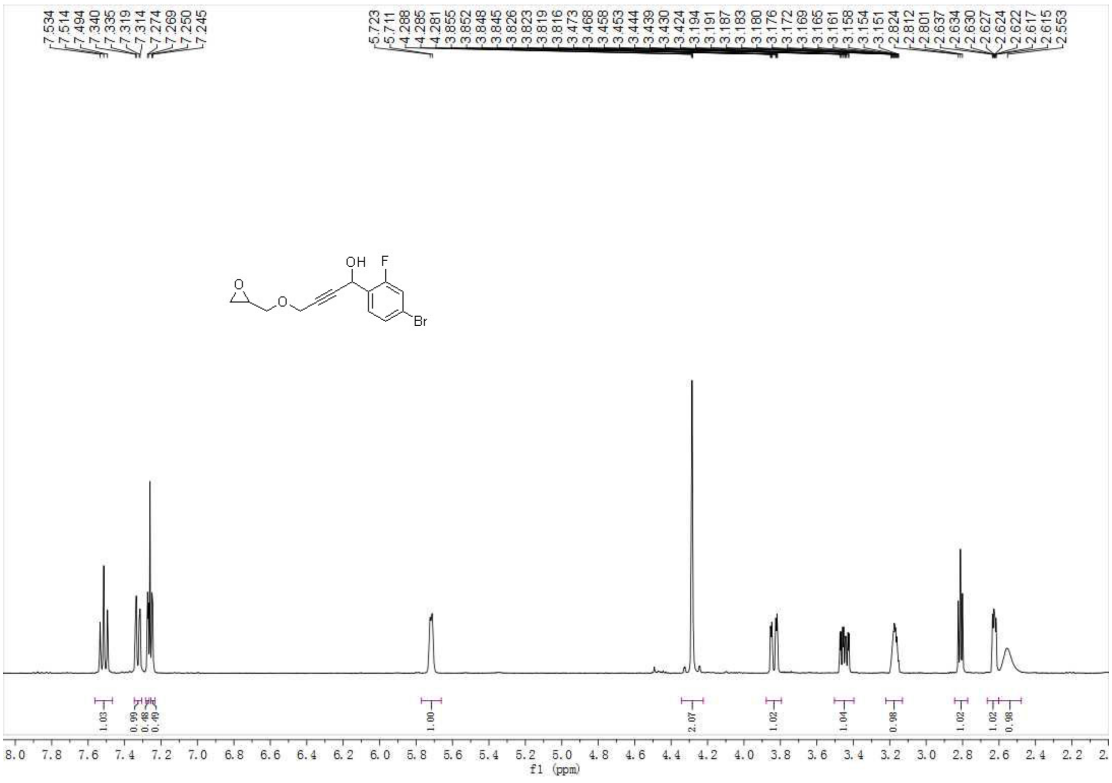

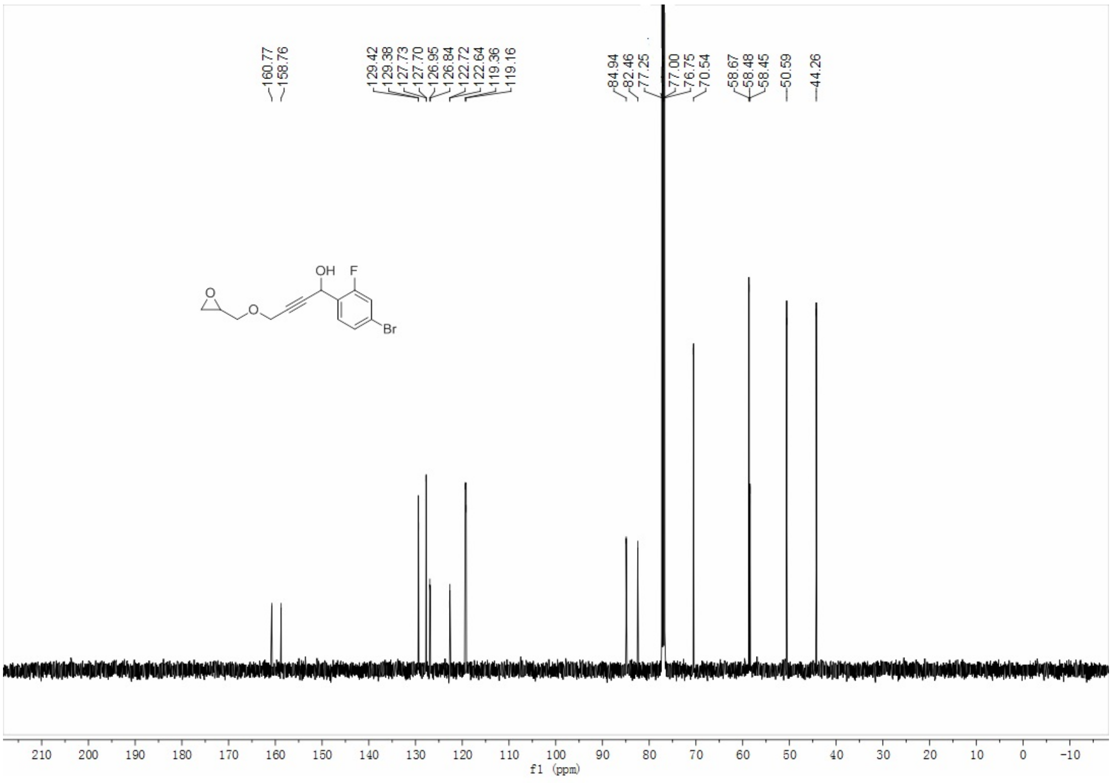

5e

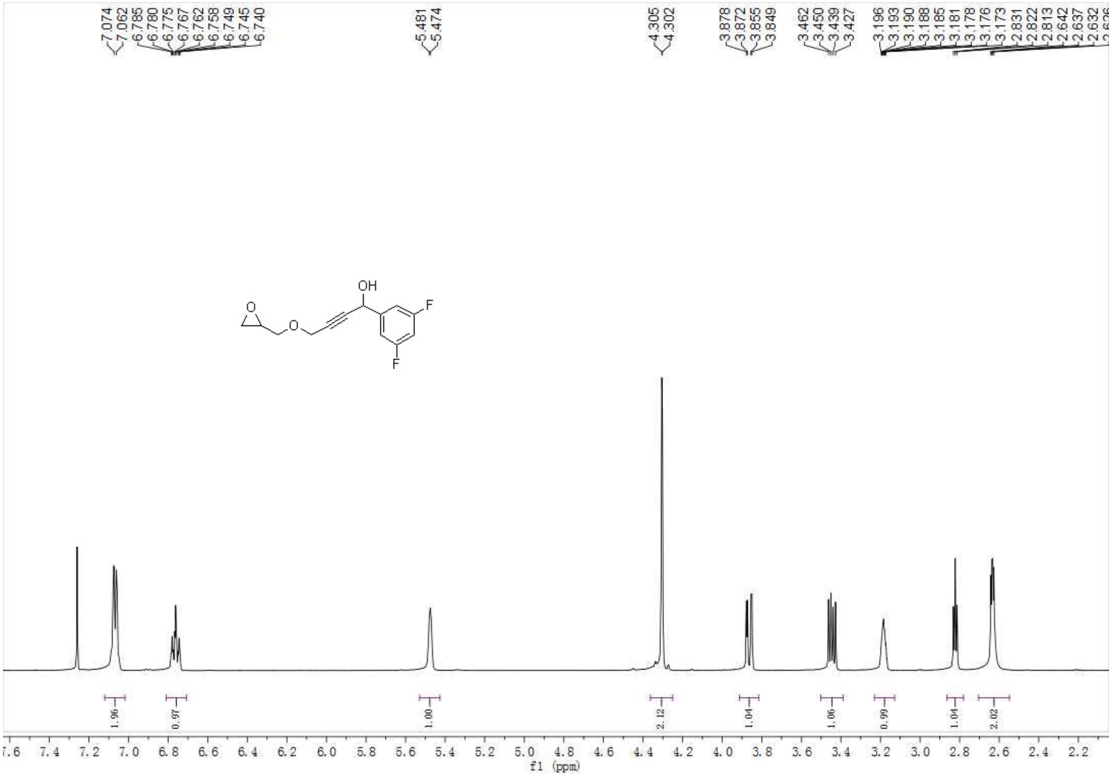
7.074
7.062
6.785
6.780
6.775
6.767
6.762
6.758
6.749
6.745
6.740
5.481
5.474
4.305
4.302
3.878
3.872
3.855
3.849
3.462
3.450
3.439
3.427
3.196
3.193
3.190
3.188
3.185
3.181
3.178
3.176
3.173
2.831
2.822
2.813
2.642
2.637
2.632
OH
O
O
F
F
1.96
0.97
1.00
2.12
1.04
1.06
0.99
1.04
2.02
f1 (ppm)

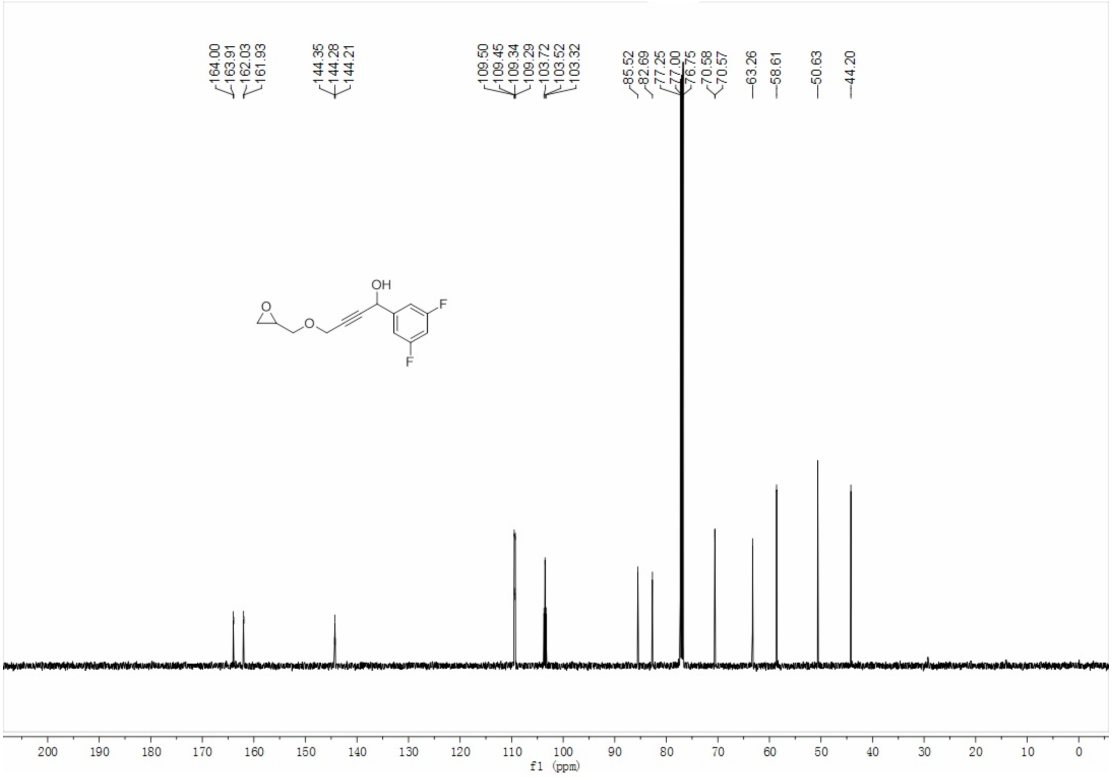
164.00
163.91
162.03
161.93
144.35
144.28
144.21
109.50
109.45
109.34
109.29
103.72
103.52
103.32
85.52
82.69
77.25
77.00
76.75
70.58
70.57
63.26
58.61
50.63
44.20
OH
O
O
F
F
f1 (ppm)

5f

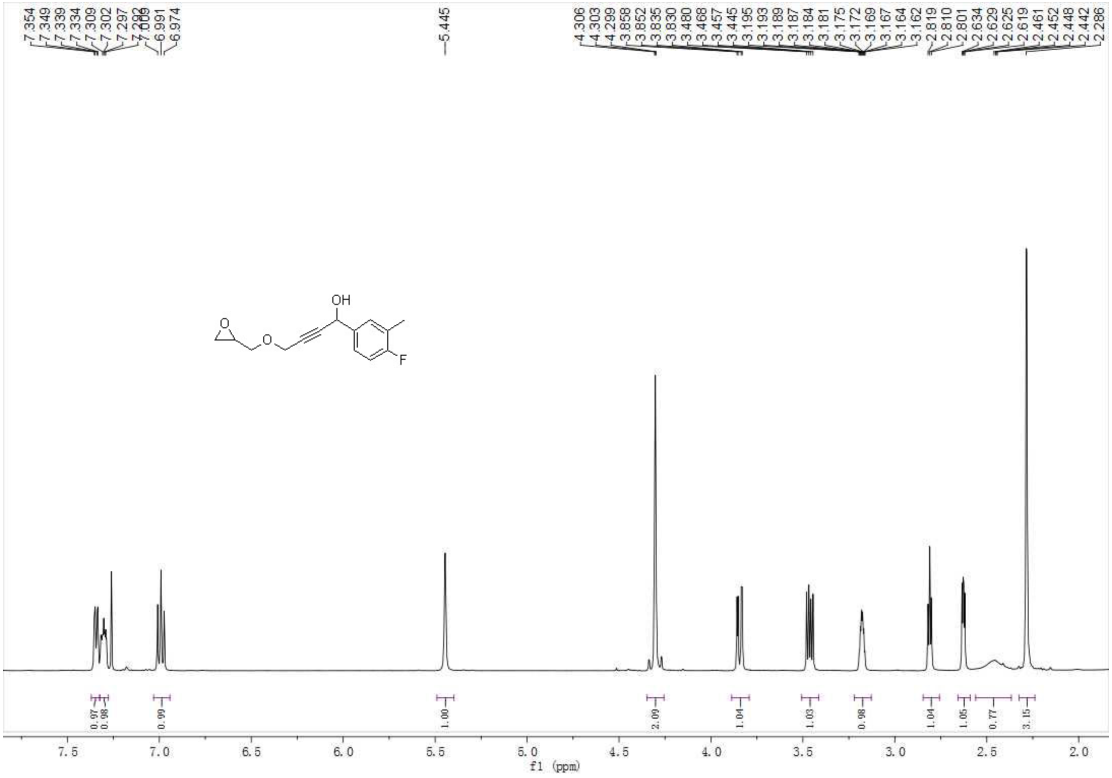

7.354
7.349
7.339
7.334
7.309
7.302
7.297
7.282
7.008
6.991
6.974
5.445
4.306
4.303
4.299
3.858
3.852
3.835
3.830
3.480
3.468
3.457
3.445
3.195
3.193
3.189
3.187
3.184
3.181
3.175
3.172
3.169
3.167
3.164
3.162
2.819
2.810
2.801
2.634
2.629
2.625
2.619
2.461
2.452
2.448
2.442
2.286
OH
O
O
F
0.97
0.98
0.99
1.00
2.09
1.04
1.03
0.98
1.04
1.05
0.77
3.15
f1 (ppm)

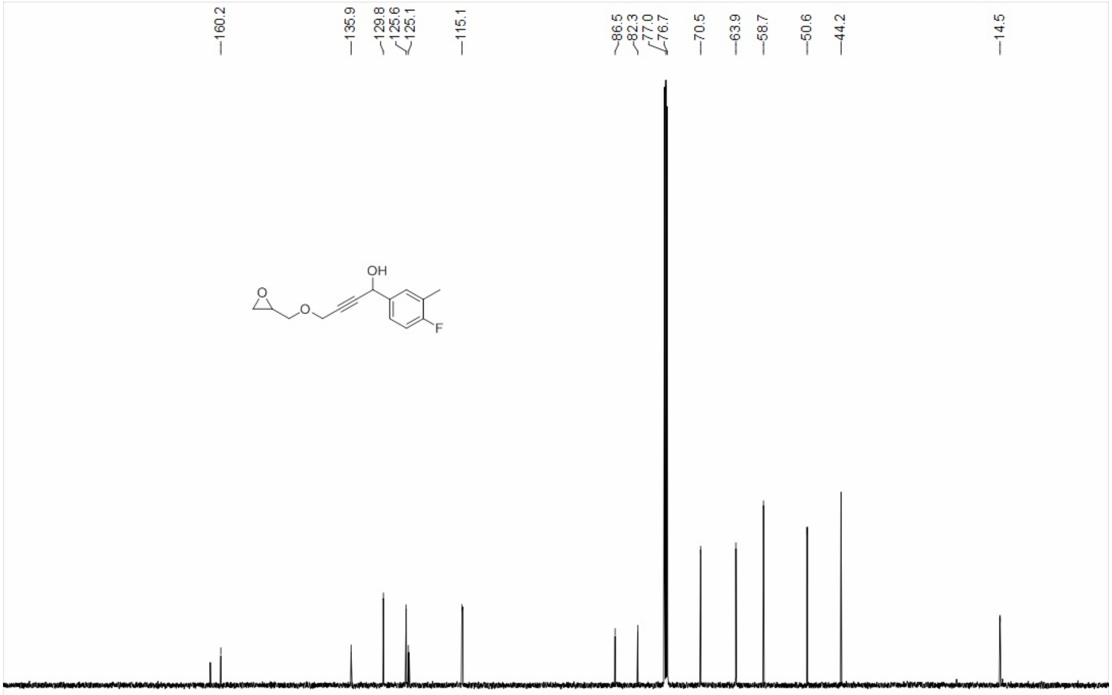

160.2
135.9
129.8
125.6
125.1
115.1
86.5
82.3
77.0
76.7
70.5
63.9
58.7
50.6
44.2
14.5
OH
O
O
F
f1 (ppm)

5g

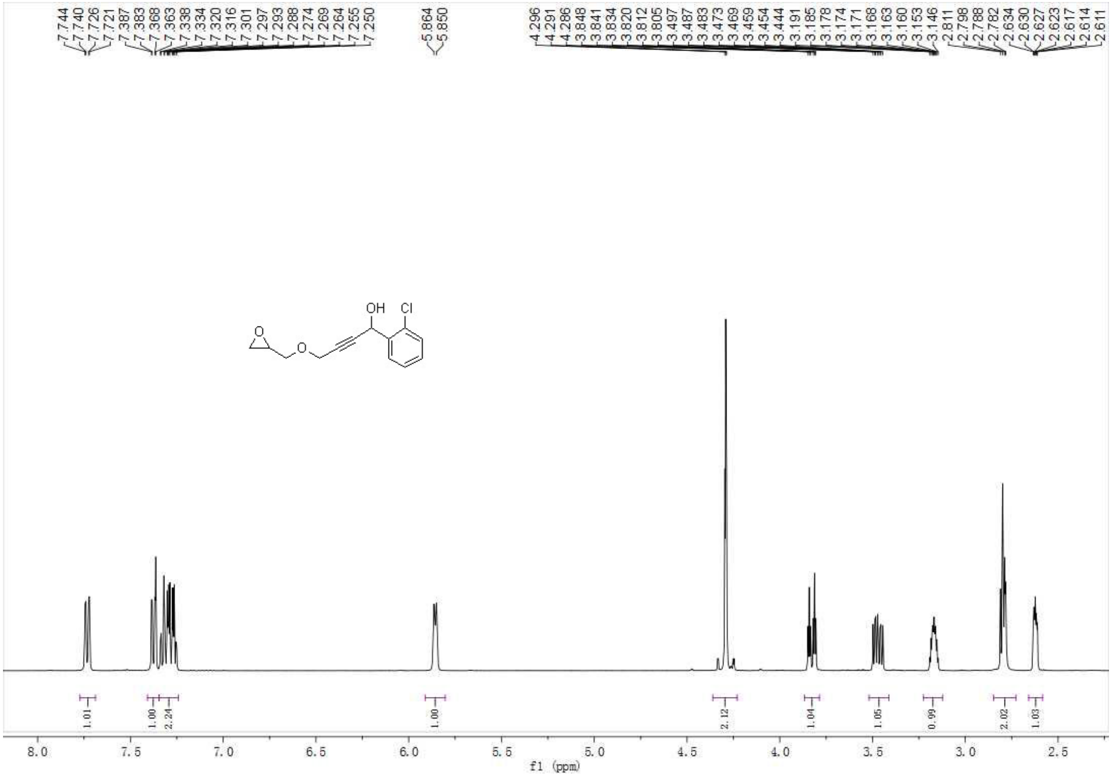
7.744
7.740
7.726
7.721
7.387
7.383
7.368
7.363
7.338
7.334
7.320
7.316
7.301
7.297
7.293
7.288
7.274
7.269
7.264
7.255
7.250
5.864
5.850
4.296
4.291
4.286
3.848
3.841
3.834
3.820
3.812
3.805
3.497
3.487
3.483
3.473
3.469
3.459
3.454
3.444
3.191
3.185
3.178
3.174
3.171
3.168
3.163
3.160
3.153
3.146
2.811
2.798
2.788
2.782
2.634
2.630
2.627
2.623
2.617
2.614
2.611
OH
Cl
O
O
1.01
1.00
2.24
1.00
2.12
1.04
1.05
0.99
2.02
1.03
f1 (ppm)

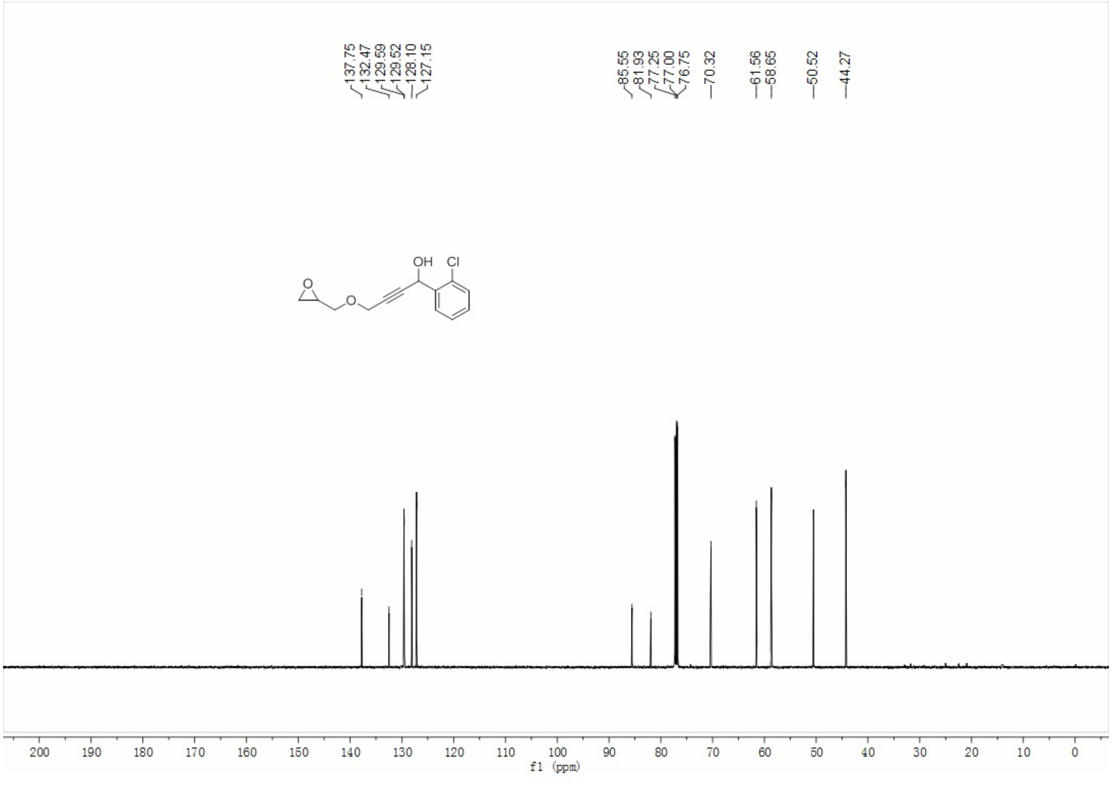
137.75
132.47
129.59
129.52
128.10
127.15
85.55
81.93
77.25
77.00
76.75
70.32
61.56
58.65
50.52
44.27
OH
Cl
O
O
f1 (ppm)

5h

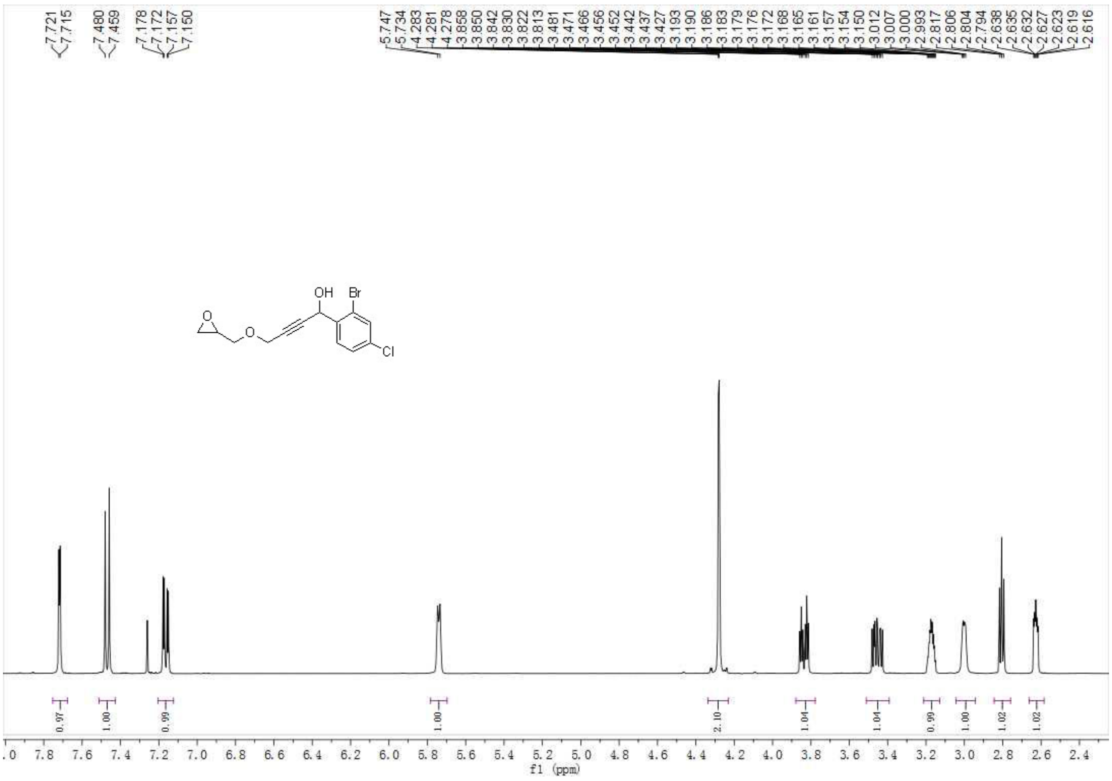

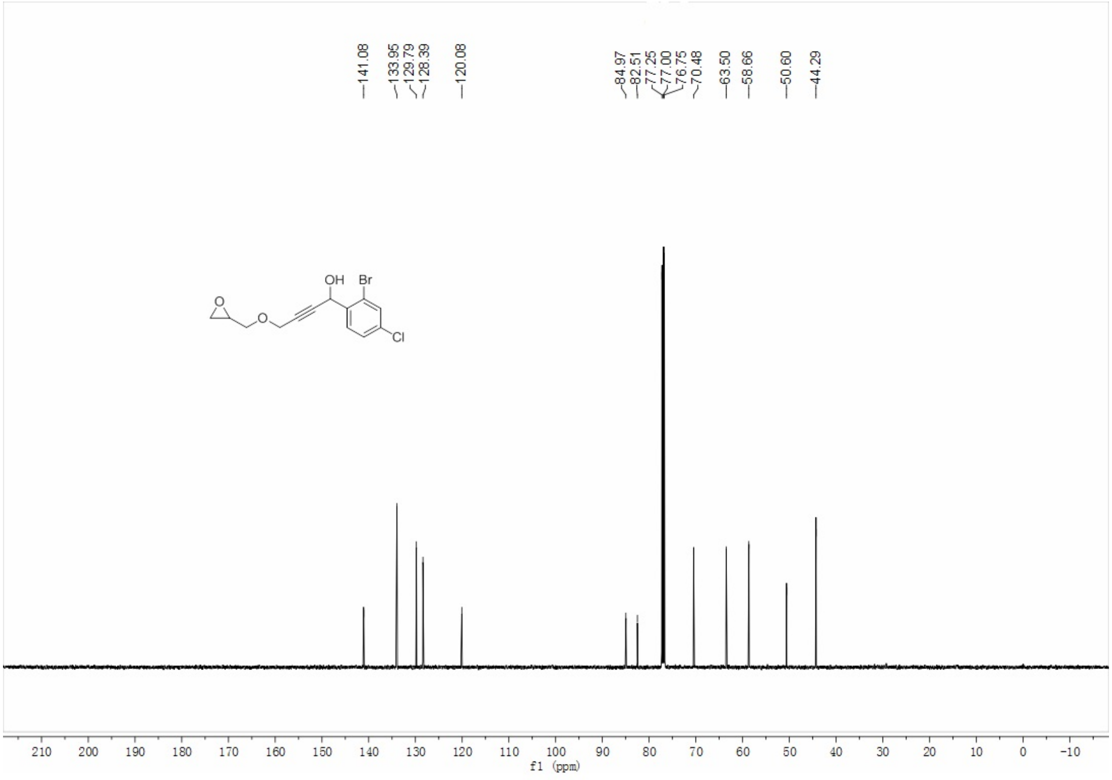

5i

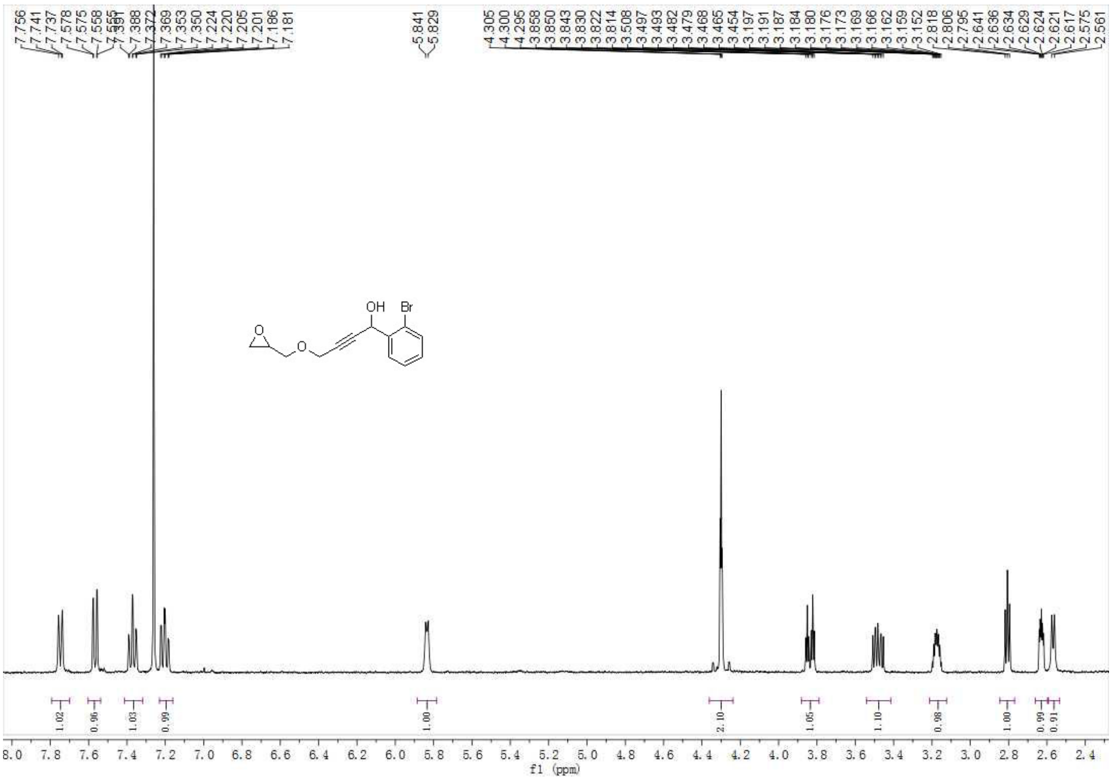

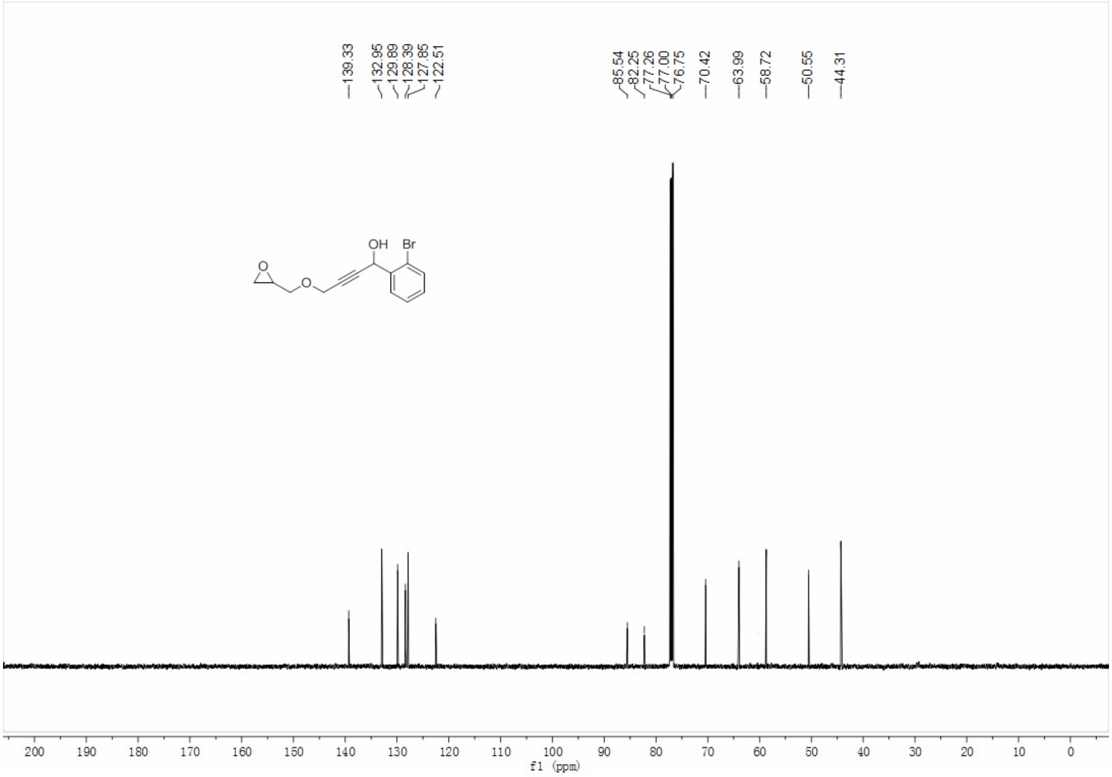

5j

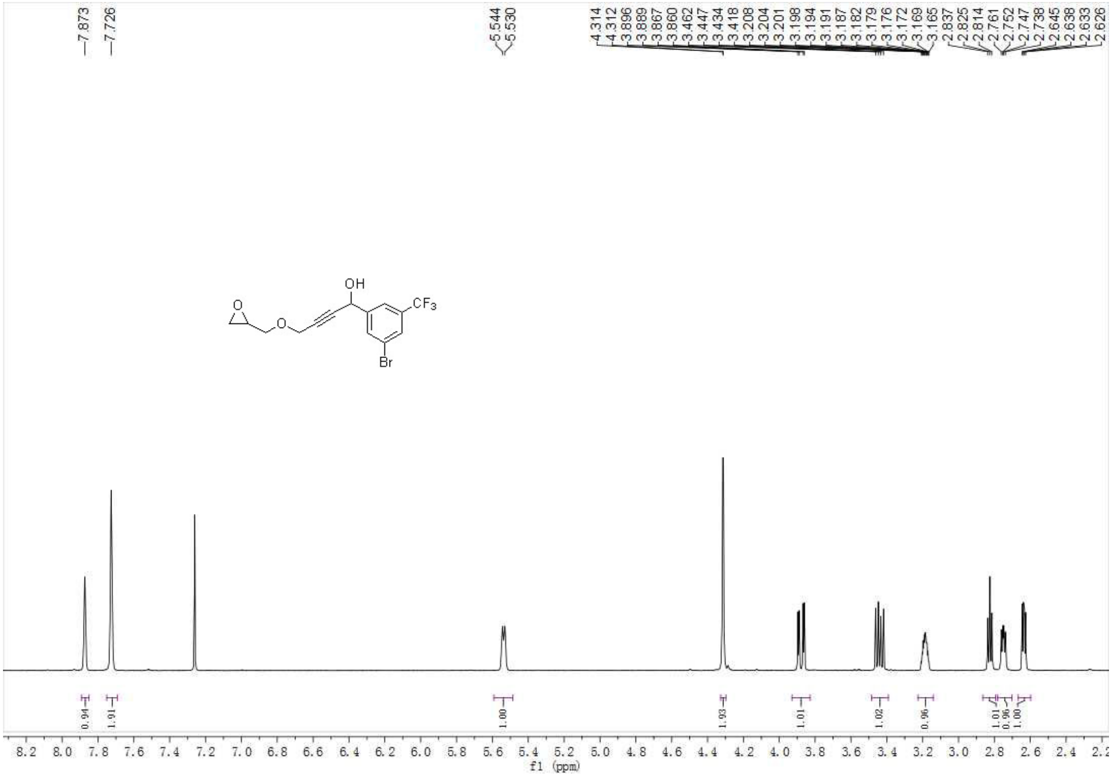

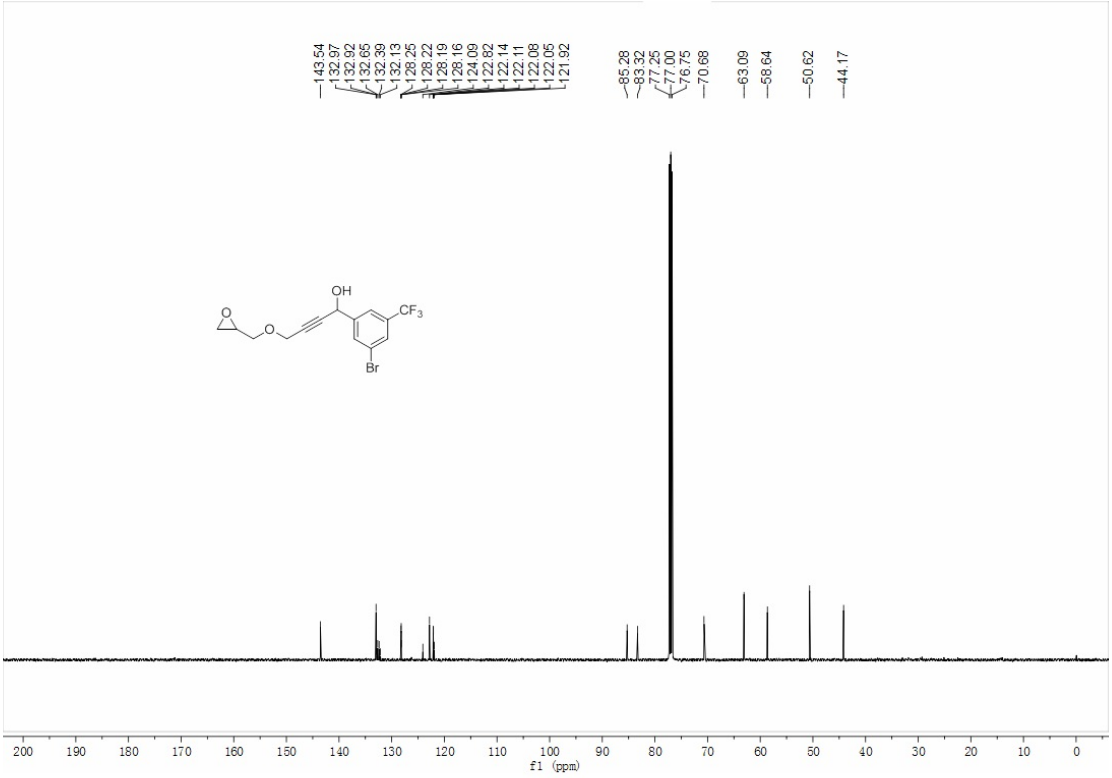

5k

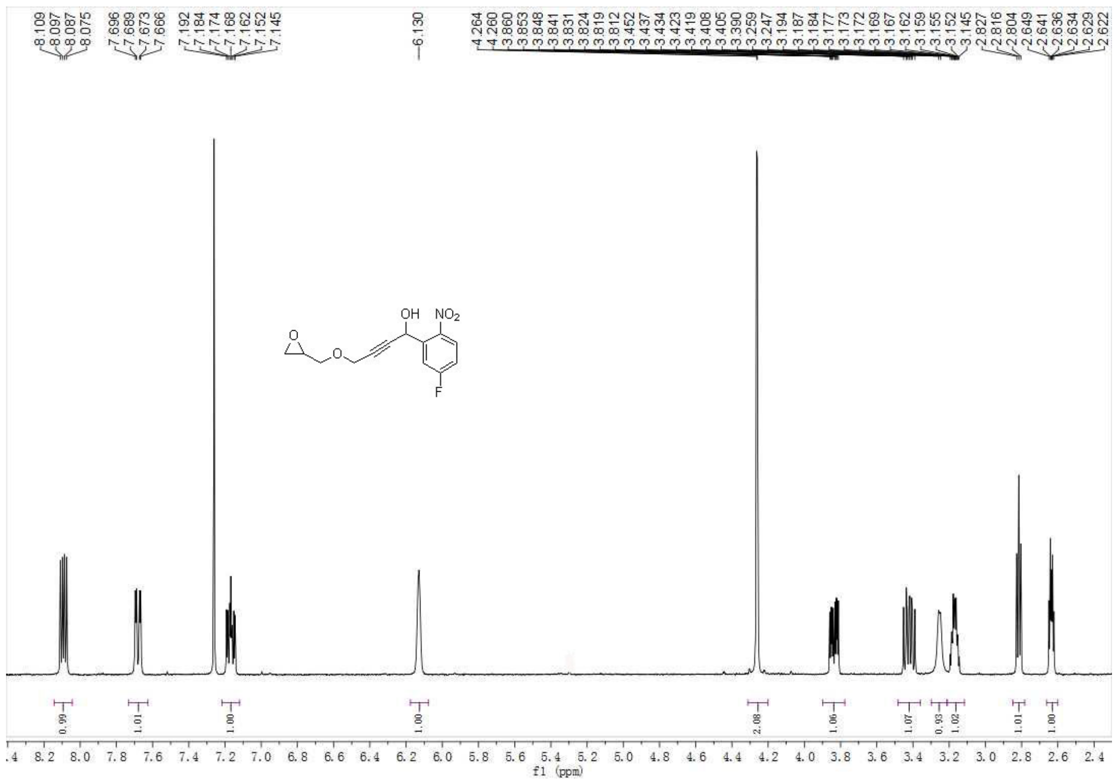

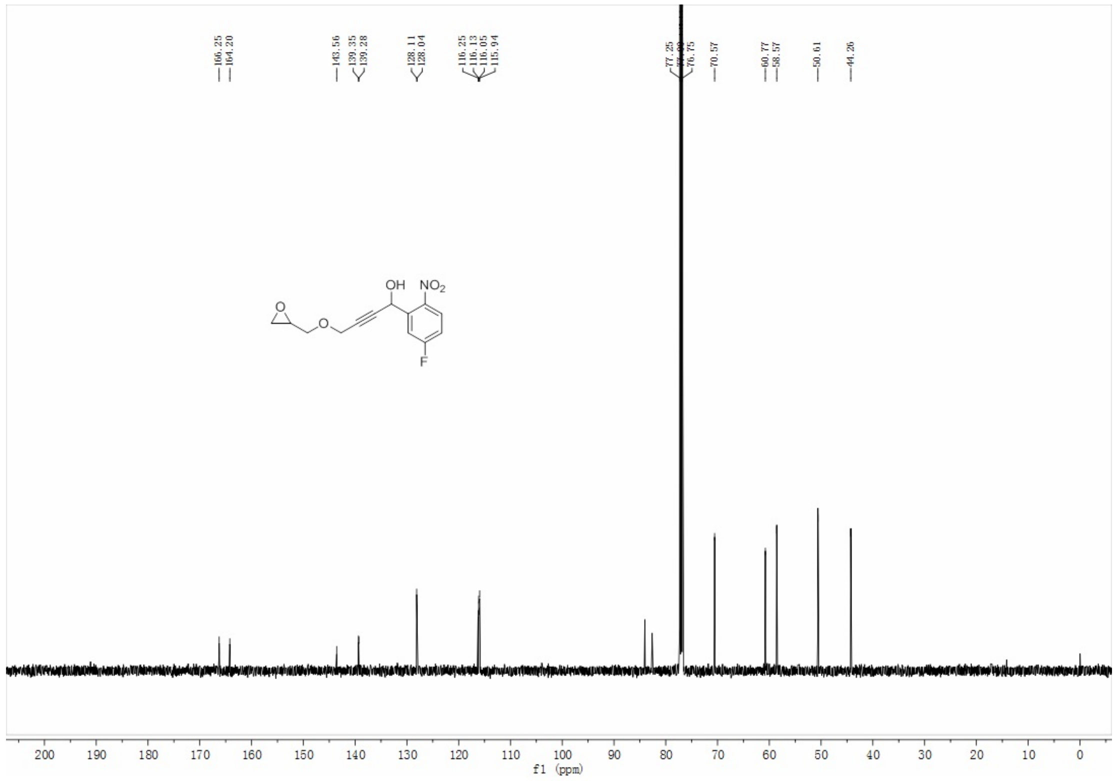

5l

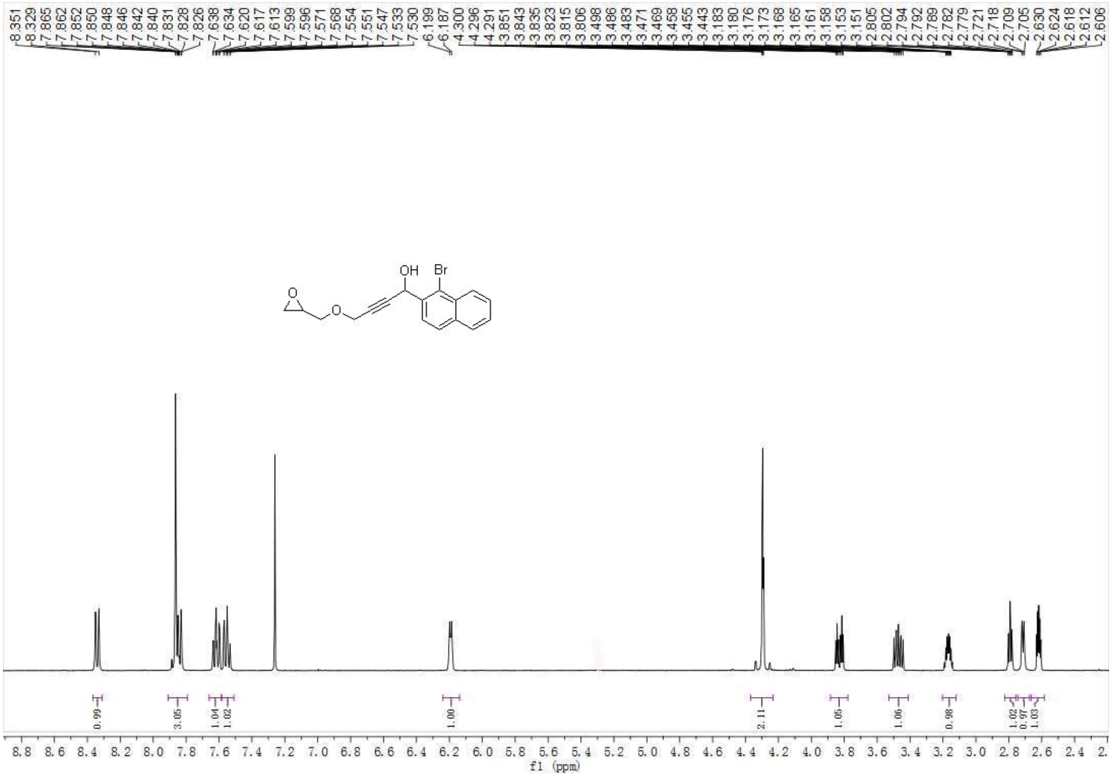

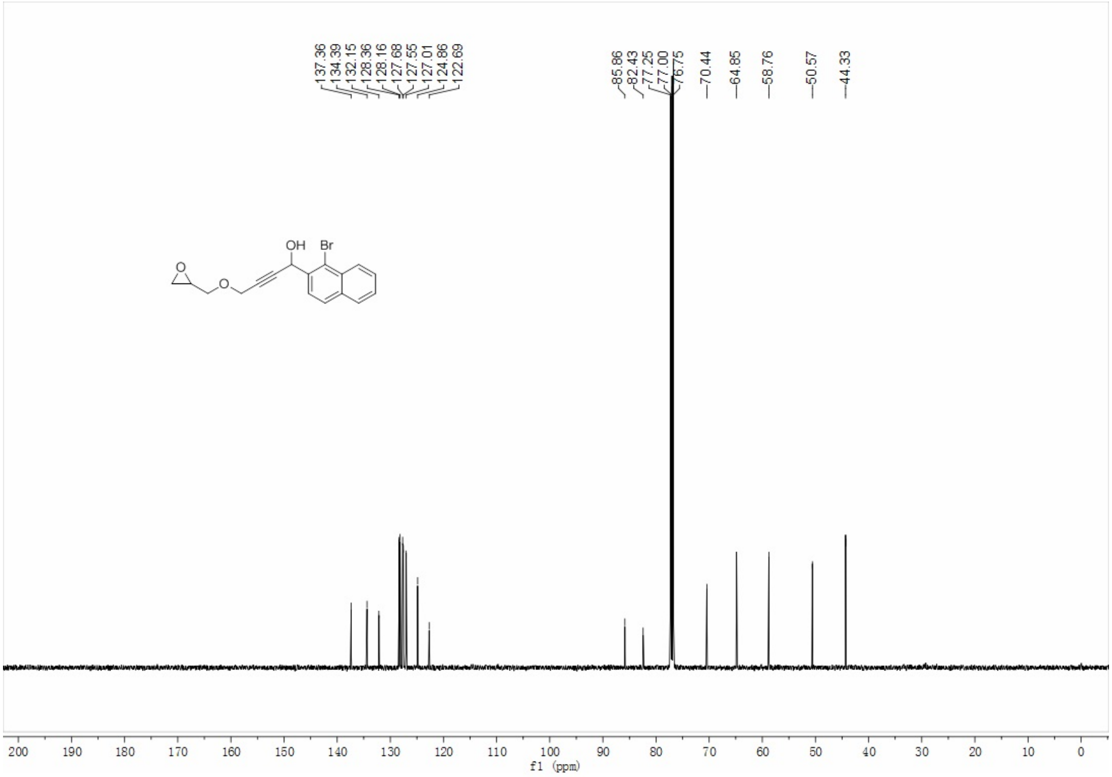

5m

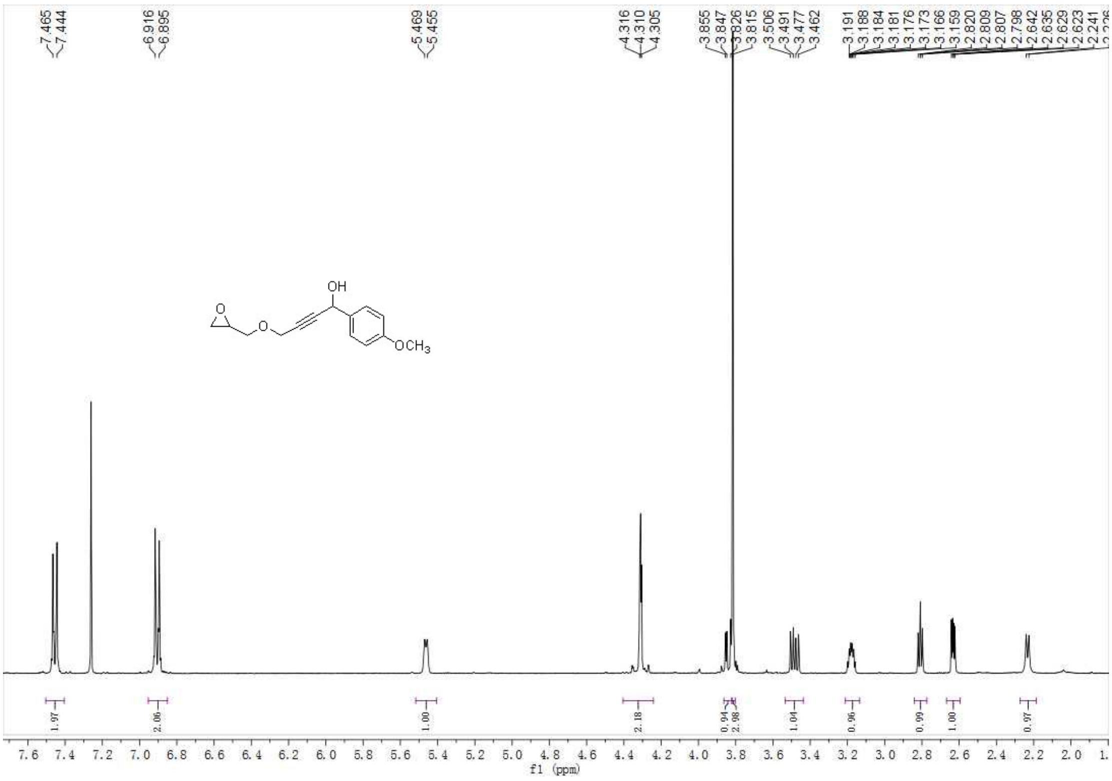
7.465
7.444
6.916
6.895
5.469
5.455
4.316
4.310
4.305
3.855
3.847
3.826
3.815
3.506
3.491
3.477
3.462
3.191
3.188
3.184
3.181
3.176
3.173
3.166
3.159
2.820
2.809
2.807
2.798
2.642
2.635
2.629
2.623
2.241
OH
O
O
OCH3
1.97
2.06
1.00
2.18
0.94
2.98
1.04
0.96
0.99
1.00
0.97
f1 (ppm)

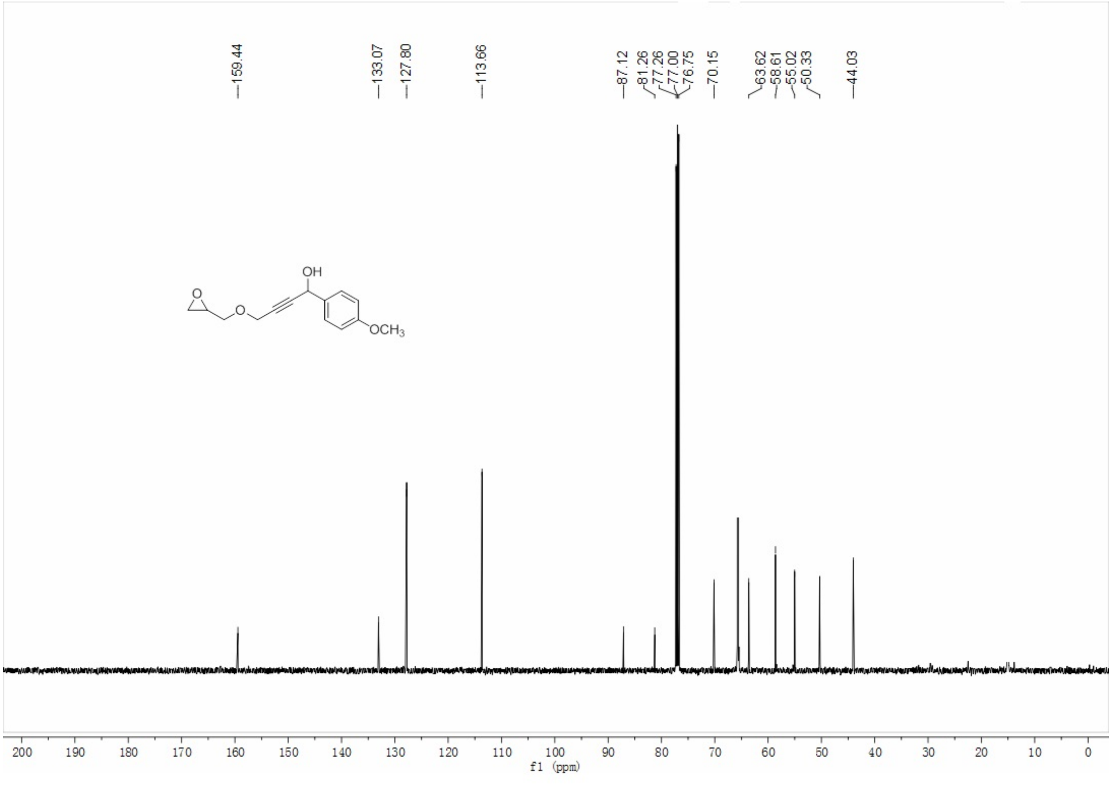
159.44
133.07
127.80
113.66
87.12
81.26
77.26
77.00
76.75
70.15
63.62
58.61
55.02
50.33
44.03
OH
O
O
OCH3
f1 (ppm)

5n

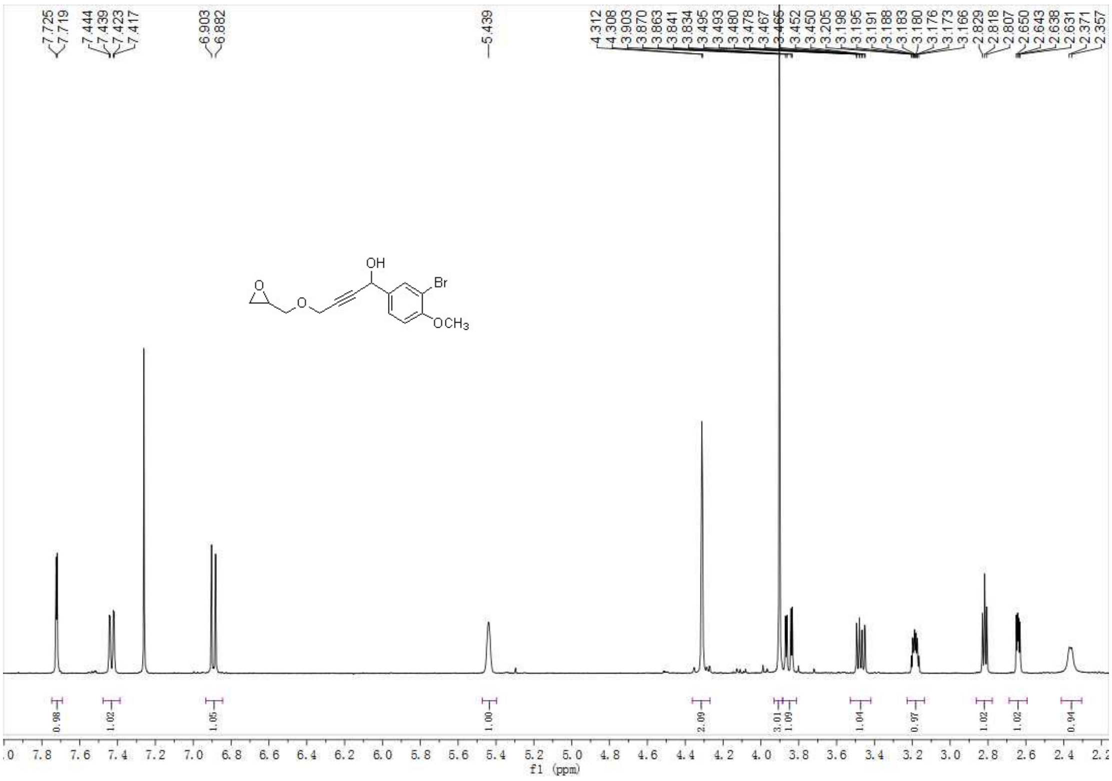

7.725
7.719
7.444
7.439
7.423
7.417
6.903
6.882
5.439
4.312
4.308
3.903
3.870
3.863
3.841
3.834
3.495
3.493
3.480
3.478
3.467
3.465
3.452
3.450
3.205
3.198
3.195
3.191
3.188
3.183
3.180
3.176
3.173
3.166
2.829
2.818
2.807
2.650
2.643
2.638
2.631
2.371
2.357
OH
Br
O
O
OCH3
0.98
1.02
1.05
1.00
2.09
3.01
1.09
1.04
0.97
1.02
1.02
0.94
f1 (ppm)

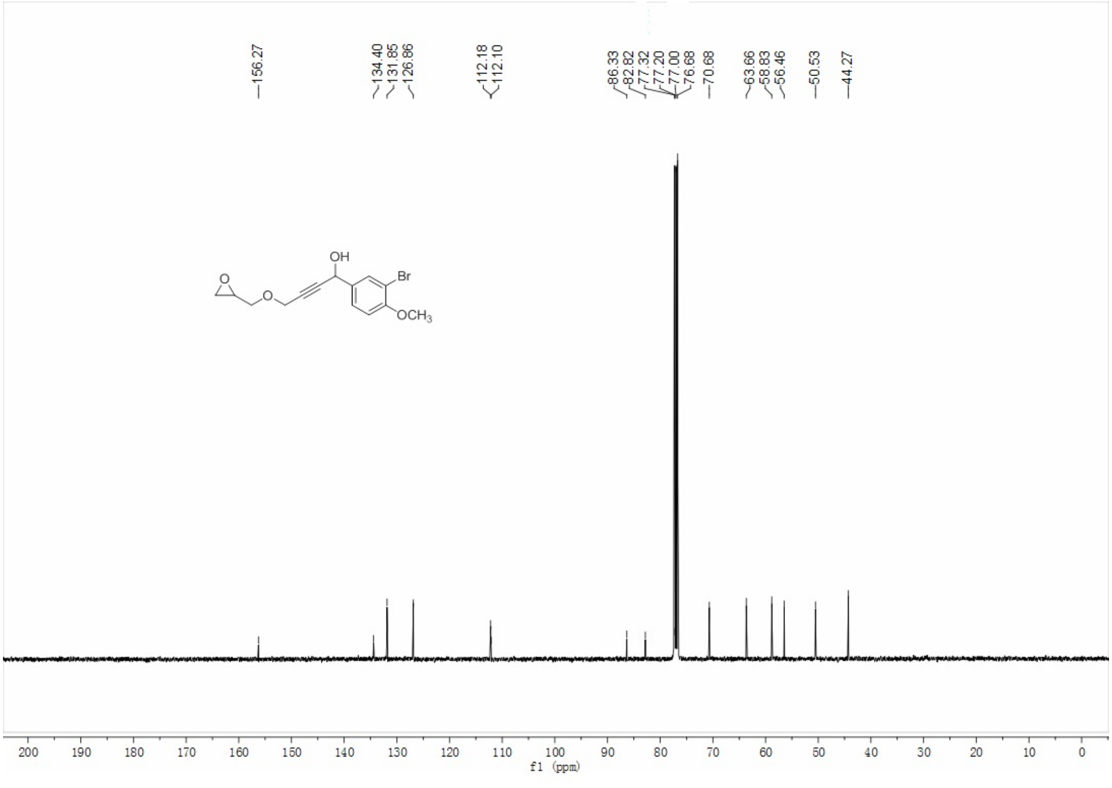

156.27
134.40
131.85
126.86
112.18
112.10
86.33
82.82
77.32
77.20
77.00
76.68
70.68
63.66
58.83
56.46
50.53
44.27
OH
Br
O
O
OCH3
f1 (ppm)

5o

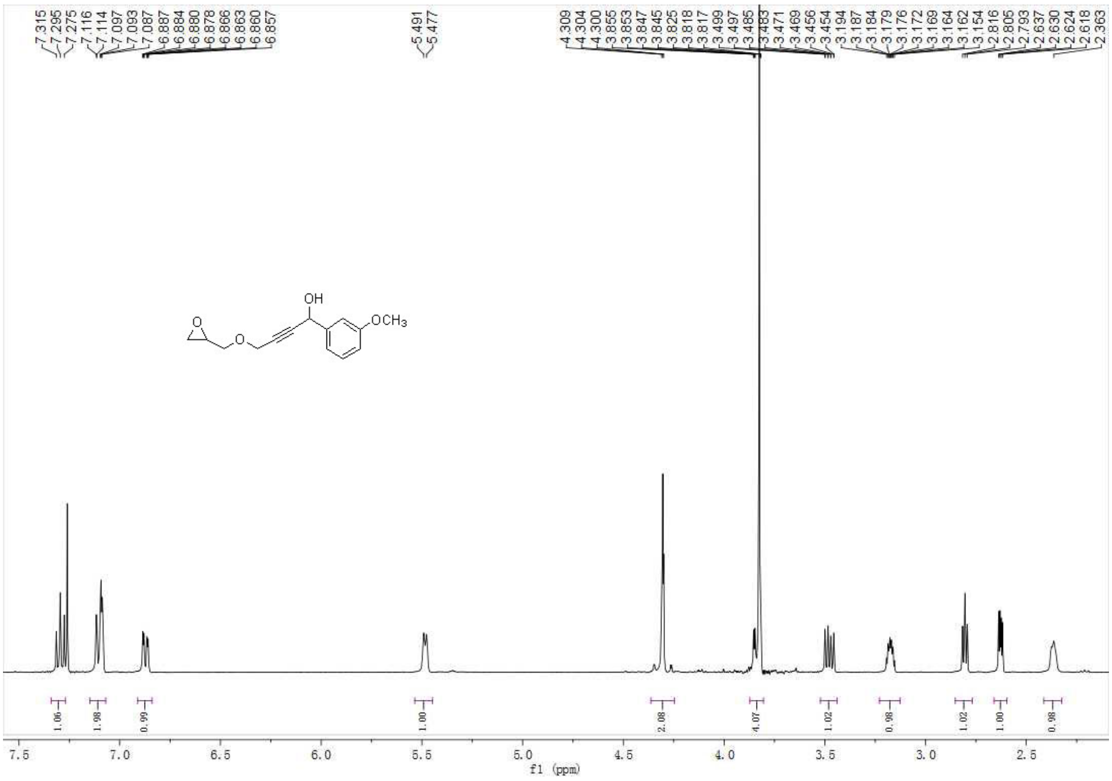

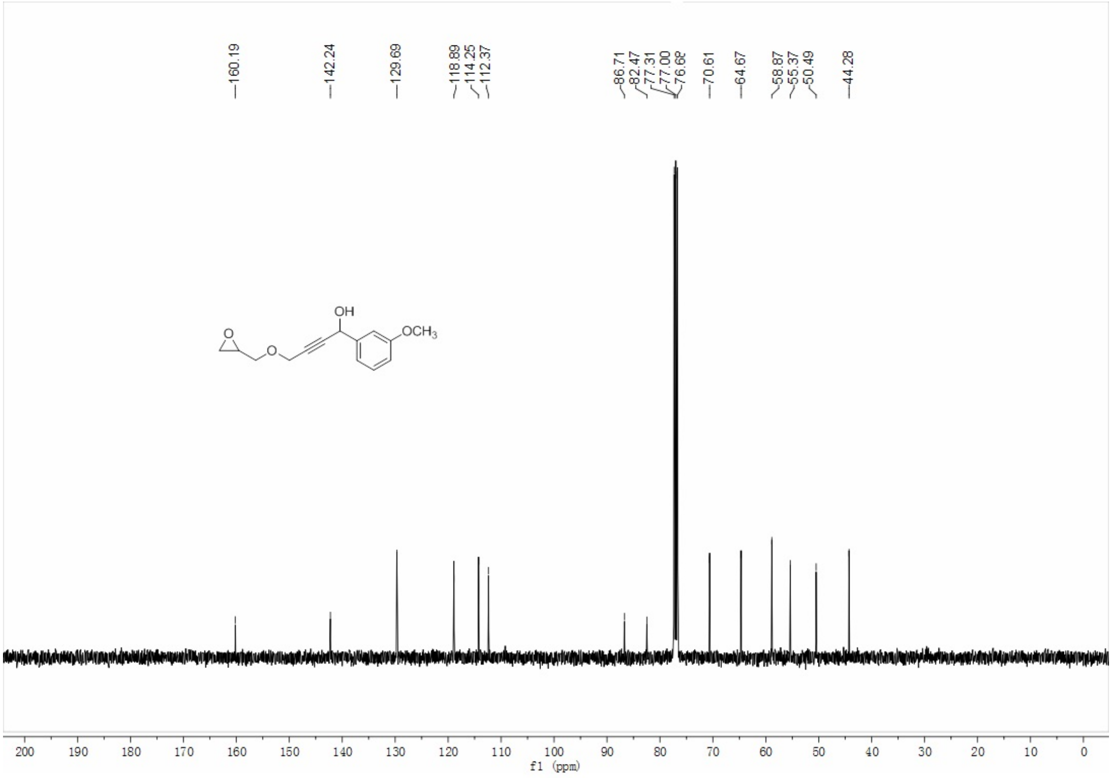

5p

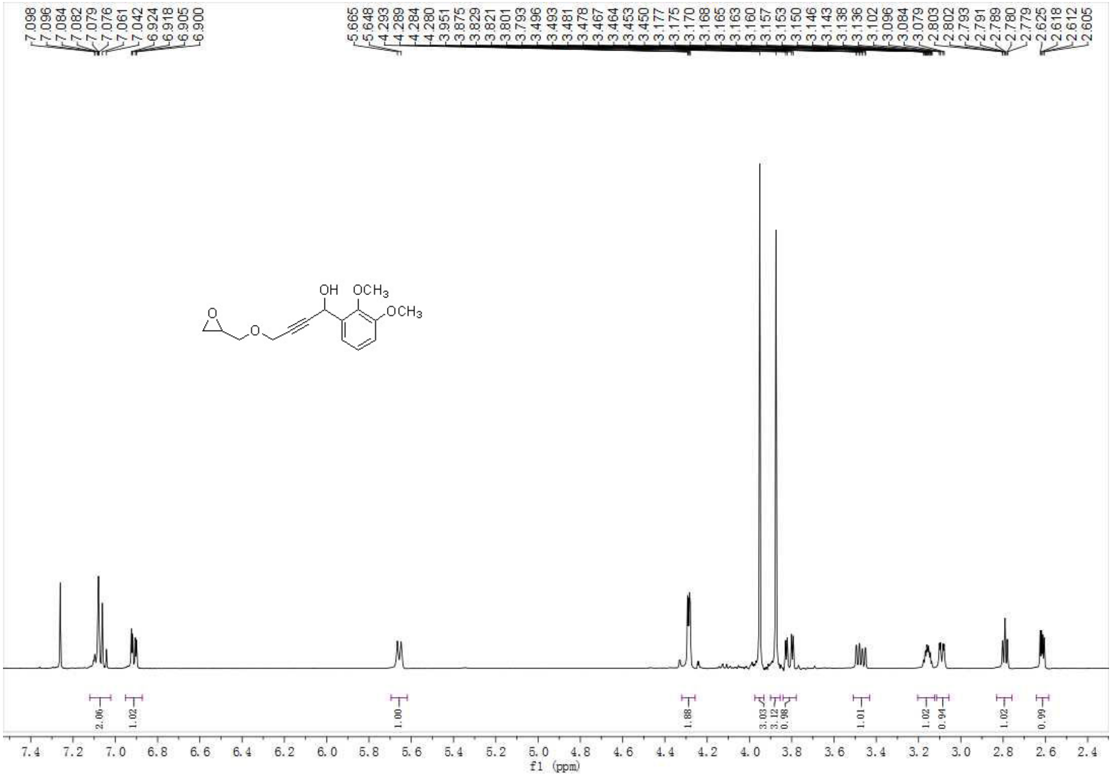
OH
OCH3
OCH3
O
f1 (ppm)

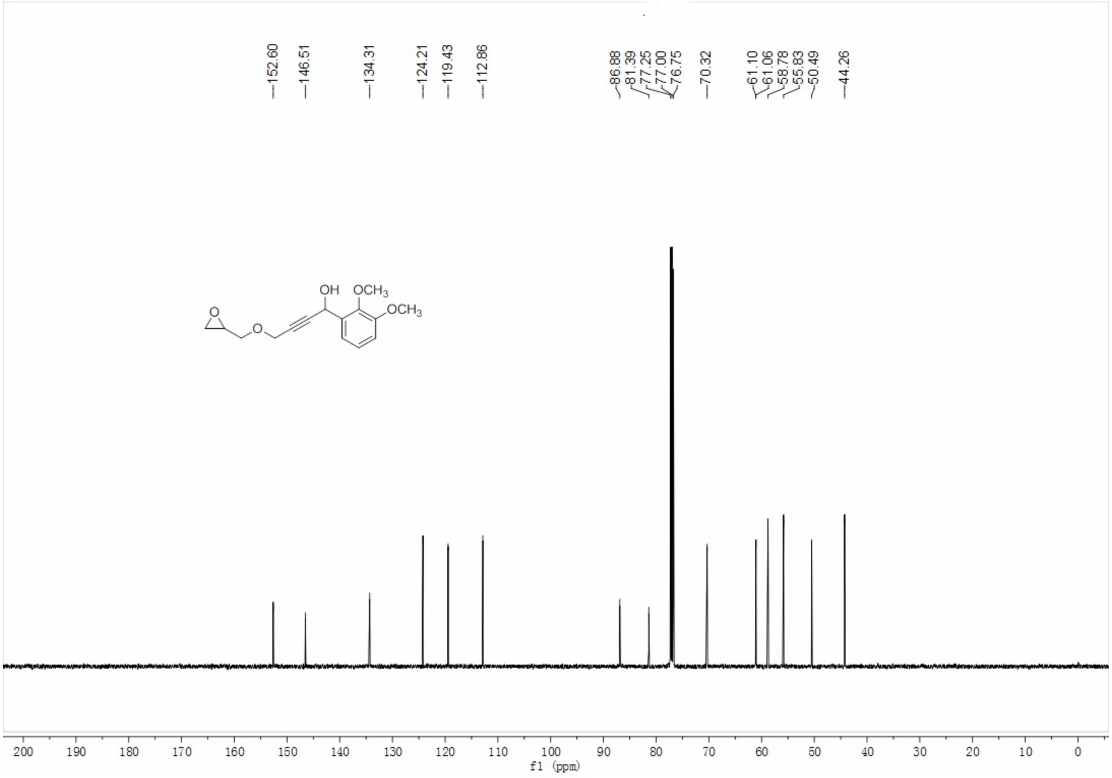
152.60
146.51
134.31
124.21
119.43
112.86
86.88
81.39
77.25
77.00
76.75
70.32
61.10
61.06
58.78
55.83
50.49
44.26
OH
OCH3
OCH3
O
f1 (ppm)

5q

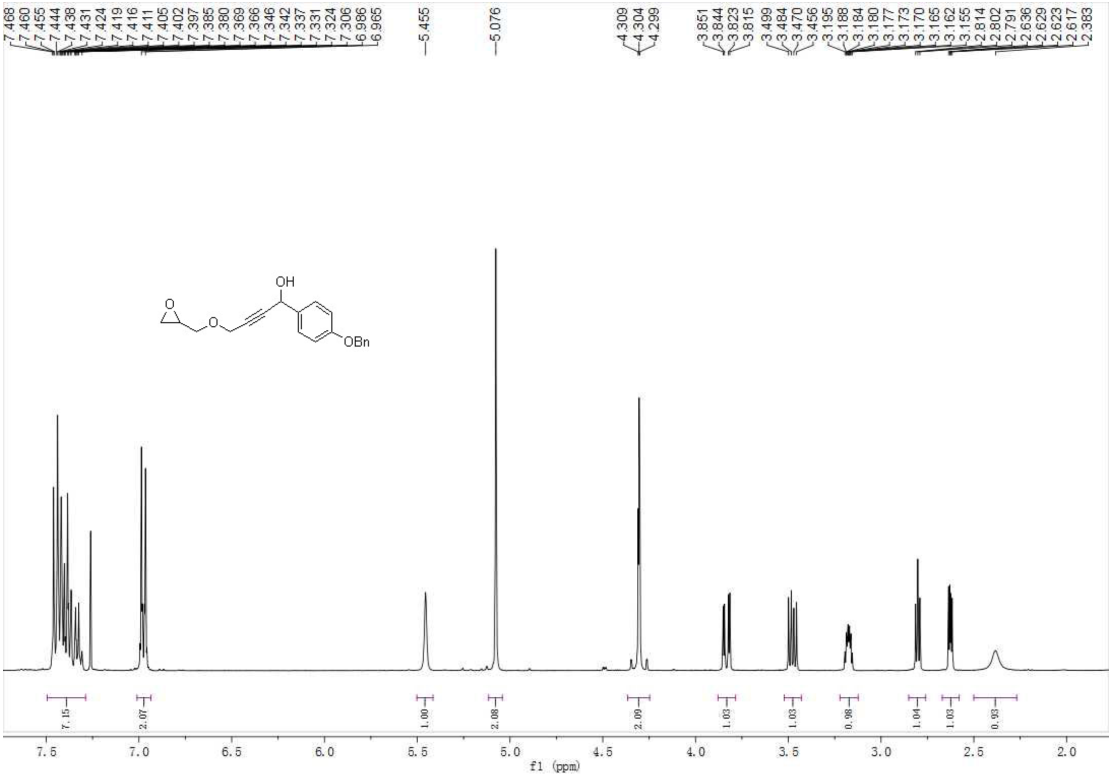
OH
O
O
OBn
f1 (ppm)

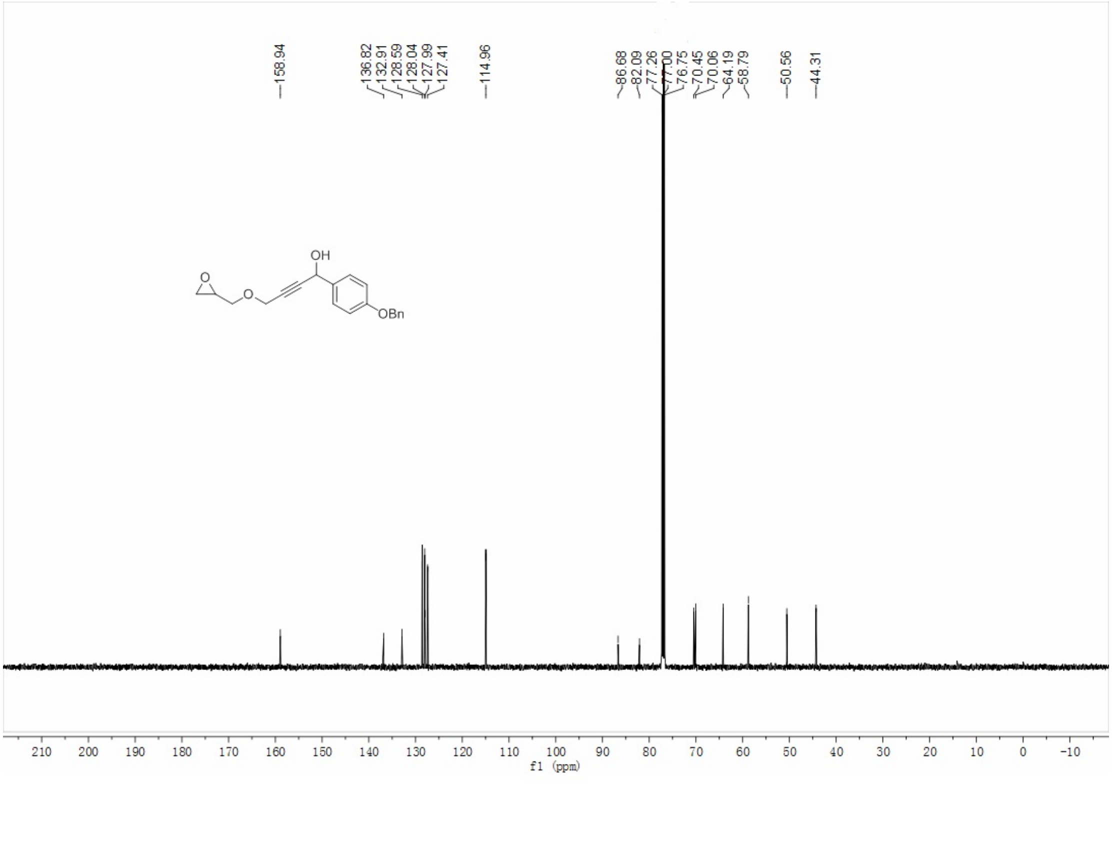
158.94
136.82
132.91
128.59
128.04
127.99
127.41
114.96
86.68
82.09
77.26
77.00
76.75
70.45
70.06
64.19
58.79
50.56
44.31
OH
O
O
OBn
f1 (ppm)

5r

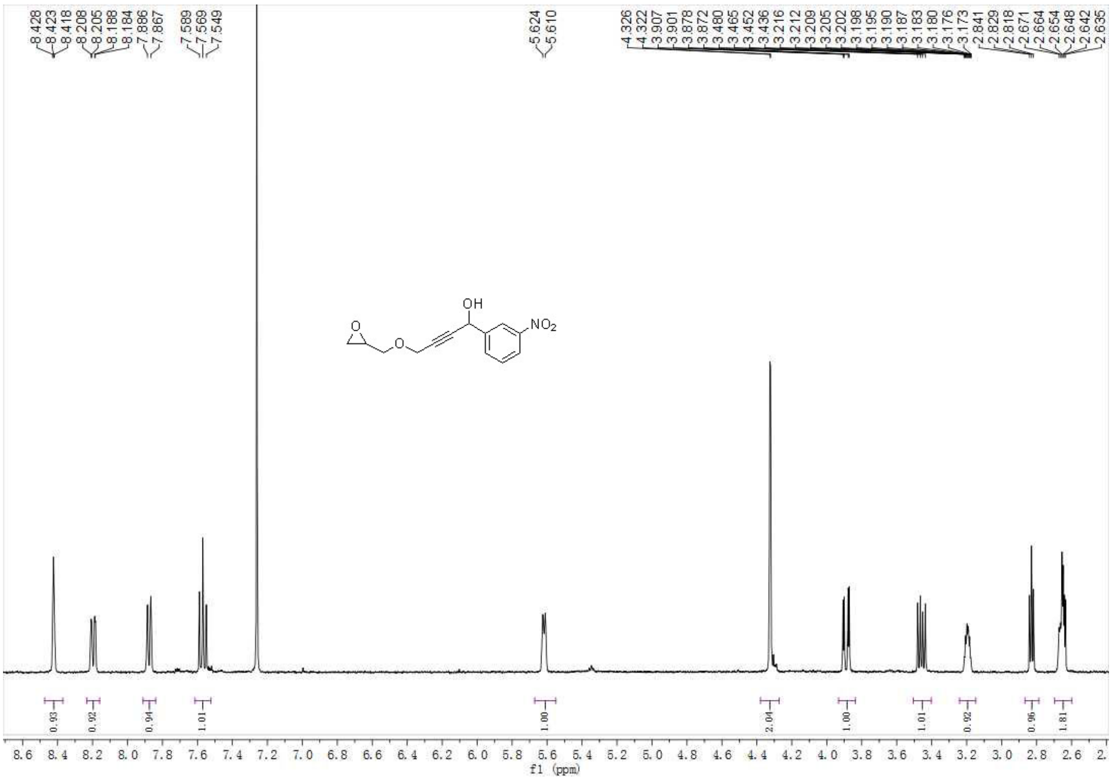

8.428
8.423
8.418
8.208
8.205
8.188
8.184
7.886
7.867
7.589
7.569
7.549
5.624
5.610
4.326
4.322
3.907
3.901
3.878
3.872
3.480
3.465
3.452
3.436
3.216
3.212
3.209
3.205
3.202
3.198
3.195
3.190
3.187
3.183
3.180
3.176
3.173
2.841
2.829
2.818
2.671
2.664
2.654
2.648
2.642
2.635
OH
NO2
O
O
0.93
0.92
0.94
1.01
1.00
2.04
1.00
1.01
0.92
0.96
1.81
f1 (ppm)

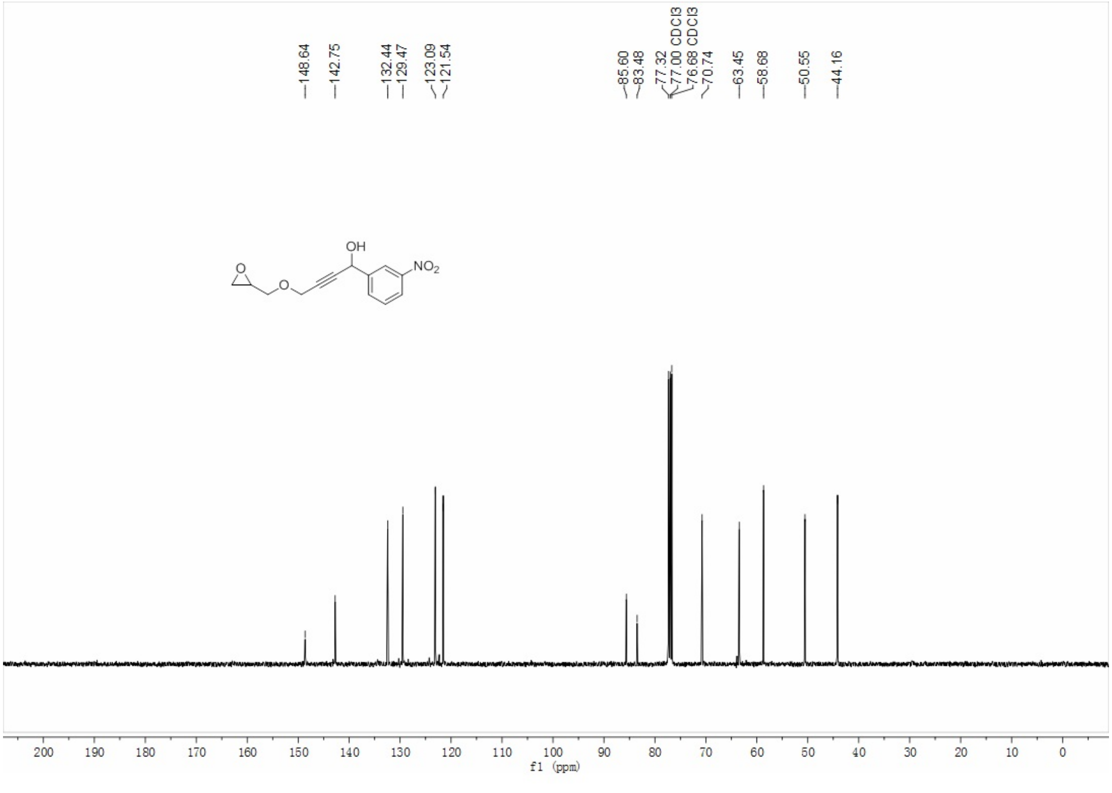

148.64
142.75
132.44
129.47
123.09
121.54
85.60
83.48
77.32 CDCl3
77.00 CDCl3
76.68 CDCl3
70.74
63.45
58.68
50.55
44.16
OH
NO2
O
O
f1 (ppm)

5s

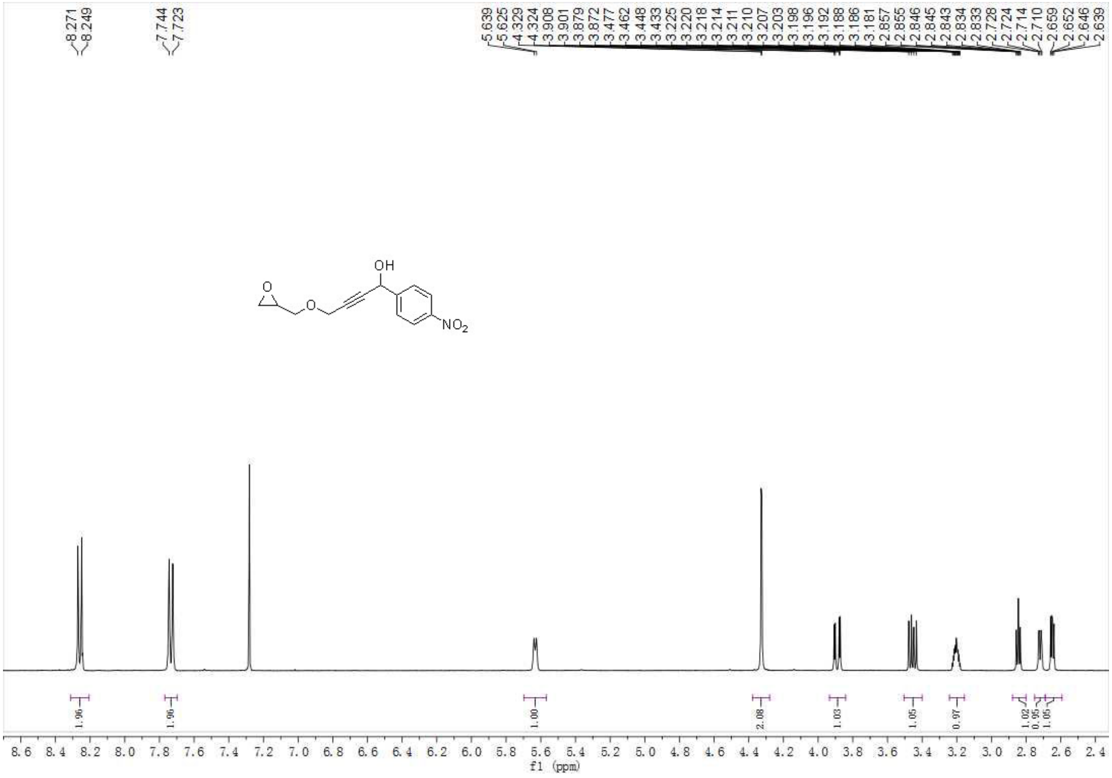

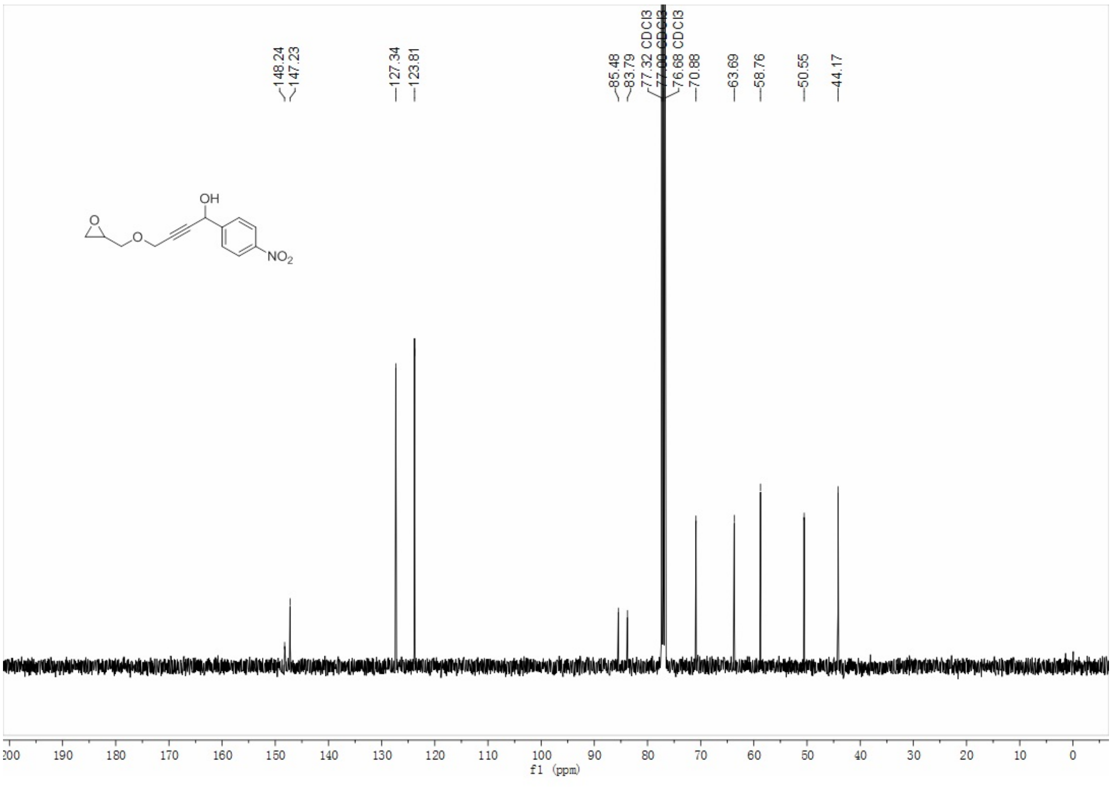

5t

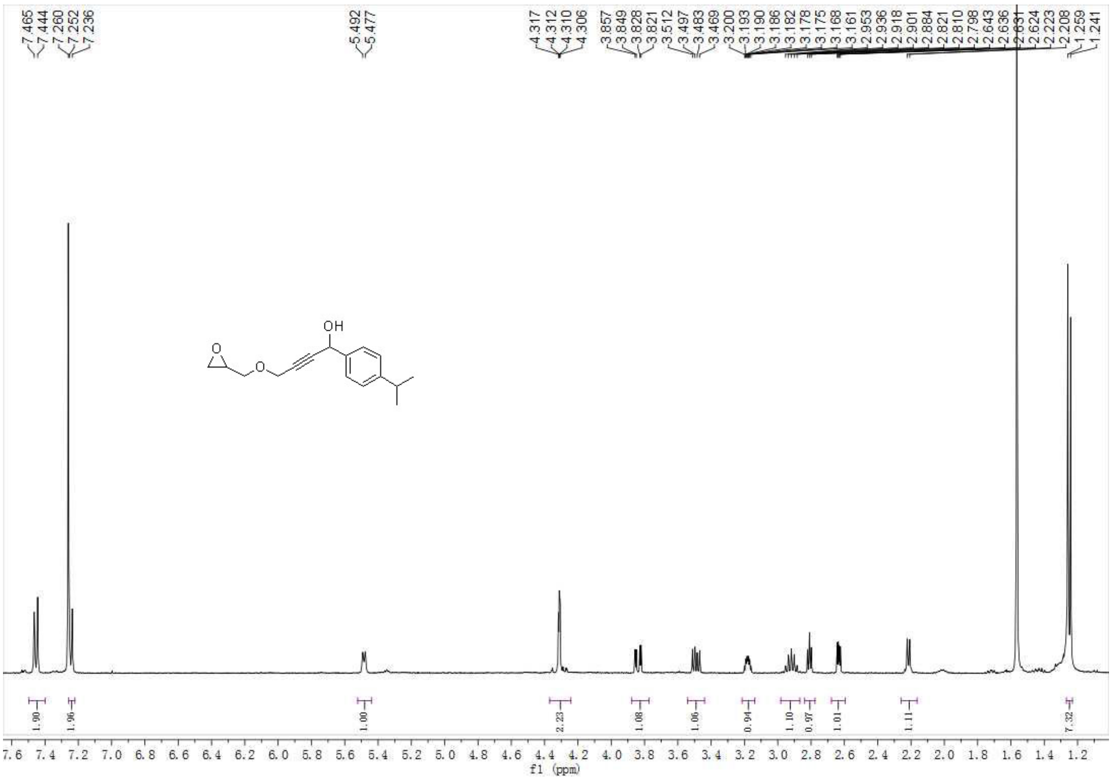

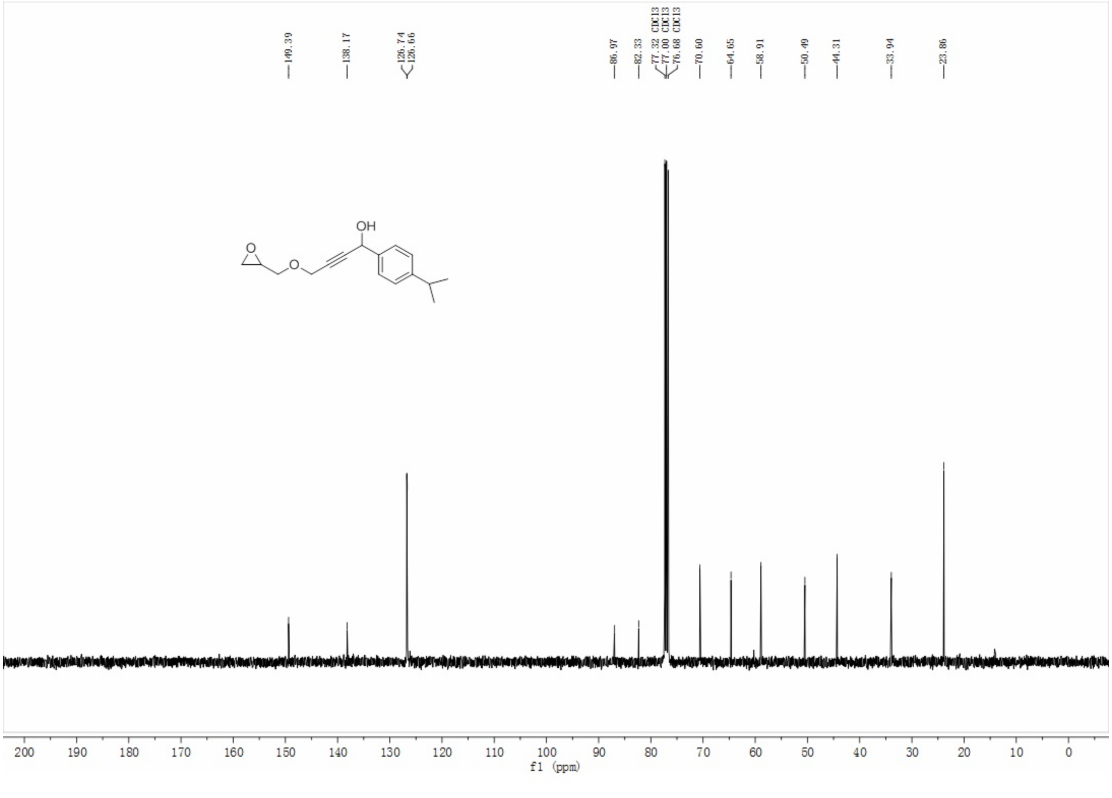

5u

3. Crystallographic Data

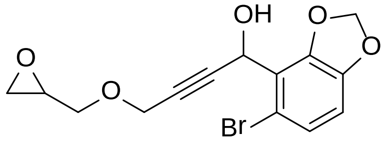

5b

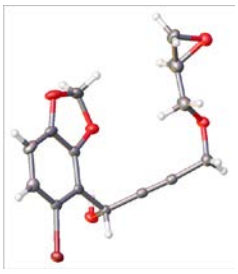

ORTEP of compound **5b**

Table S2. Crystal data and structure refinement for 5b

Identification code	**5b**
Empirical formula	$C_{28}H_{28}O_{11}Br$
Formula weight	620.41
Temperature/K	108.0(3)
Crystal system	monoclinic
Space group	$P2_1/c$
a/Å	8.15581(18)
b/Å	20.5484(3)
c/Å	8.7485(2)
α/°	90.00
β/°	115.872(3)
γ/°	90.00
Volume/Å^3	1319.20(5)
Z	2
ρ_{calc}mg/mm^3	1.562
m/mm^{-1}	2.680
F(000)	638.0
Crystal size/mm^3	0.2246 × 0.1424 × 0.0653
2Θ range for data collection	8.6 to 124.76°
Index ranges	$-9 \le h \le 9$, $-23 \le k \le 23$, $-10 \le l \le 9$

Reflections collected	8567
Independent reflections	2097[R(int) = 0.0243]
Data/restraints/parameters	2097/0/182
Goodness-of-fit on F^2	1.204
Final R indexes [I>=2σ (I)]	R_1 = 0.0368, wR_2 = 0.0777
Final R indexes [all data]	R_1 = 0.0385, wR_2 = 0.0783
Largest diff. peak/hole / e $Å^{-3}$	1.11/-0.33

Table S3. Fractional Atomic Coordinates (×10^4) and Equivalent Isotropic Displacement Parameters ($Å^2$×10^3) for 8b. U_{eq} is defined as 1/3 of of the trace of the orthogonalised U_{IJ} tensor.

**Atom**	***x***	***y***	***z***	**U(eq)**
Br1	1077.8(5)	4574.61(18)	2356.0(5)	20.47(14)
O1	8692(3)	6758.5(13)	2423(3)	23.1(6)
O2	1476(3)	6275.1(13)	483(3)	21.9(6)
C3	3817(5)	5650.0(19)	6751(5)	24.8(9)
O5	4912(4)	6792.4(13)	4259(3)	25.3(6)
O6	11787(4)	7353.4(14)	5686(3)	27.8(6)
O7	5470(4)	6683.5(13)	7093(3)	30.7(7)
C8	4126(5)	6220.6(17)	4496(5)	19.0(8)
C9	6032(5)	6077.0(17)	1281(5)	18.0(8)
C10	4624(5)	5977.0(17)	1351(5)	17.8(8)
C11	2892(5)	5828.9(17)	1440(5)	17.2(8)
C12	4446(5)	6163.2(18)	6177(5)	22.3(9)
C13	3169(5)	5764.4(17)	3268(5)	17.0(8)
C14	2495(5)	5243.4(17)	3870(5)	17.3(8)
C15	5645(6)	7111(2)	5877(6)	31.7(10)
C16	7813(5)	6228.4(19)	1315(5)	22.5(8)
C17	2804(5)	5181.7(18)	5550(5)	22.7(9)
C19	11311(6)	7100(2)	6991(5)	27.4(9)
C20	9940(6)	7158(2)	5257(6)	37.2(11)
C22	9255(6)	6574(2)	4164(5)	30.6(10)

Table S4. Anisotropic Displacement Parameters ($Å^2$×10^3) for 8b. The Anisotropic displacement factor exponent takes the form: $-2\pi^2[h^2a^{*2}U_{11}+...+2hka\times b\times U_{12}]$

**Atom**	U_{11}	U_{22}	U_{33}	U_{23}	U_{13}	U_{12}

Br1	20.1(2)	17.1(2)	22.1(2)	-0.56(16)	7.30(16)	-3.28(16)
O1	22.6(14)	26.3(14)	18.8(14)	-0.5(11)	7.7(12)	-7.5(11)
O2	15.4(13)	22.6(14)	21.9(14)	1.5(11)	2.7(11)	-0.6(11)
C3	32(2)	27(2)	14.2(19)	1.7(16)	8.0(17)	8.1(18)
O5	24.8(14)	25.7(15)	24.0(15)	-6.3(12)	9.2(12)	-6.2(12)
O6	25.0(14)	31.8(16)	26.3(15)	0.1(12)	10.7(12)	-3.1(12)
O7	33.1(16)	24.8(15)	25.0(15)	-11.2(12)	4.1(13)	-2.5(12)
C8	16.9(18)	14.3(18)	25(2)	-1.3(15)	8.3(16)	0.5(15)
C9	22(2)	15.2(18)	14.8(19)	-0.3(15)	6.1(16)	0.7(15)
C10	22(2)	14.8(18)	14.8(18)	-1.0(14)	6.2(16)	0.3(15)
C11	18.6(19)	13.6(18)	18.3(19)	-0.4(15)	6.9(16)	1.2(14)
C12	18.9(19)	22(2)	22(2)	-6.1(16)	5.2(17)	5.4(16)
C13	12.7(18)	17.4(19)	20.5(19)	-2.4(15)	6.8(16)	2.5(14)
C14	14.5(18)	13.7(17)	21.7(19)	-1.3(15)	6.1(15)	3.8(14)
C15	28(2)	30(2)	40(3)	-17(2)	18(2)	-11.4(18)
C16	20(2)	24(2)	24(2)	0.4(16)	10.4(17)	-0.3(16)
C17	27(2)	17.2(19)	24(2)	6.4(16)	10.9(18)	6.5(16)
C19	35(2)	29(2)	22(2)	-0.3(18)	16.0(19)	1.1(18)
C20	32(2)	48(3)	32(3)	1(2)	14(2)	-7(2)
C22	30(2)	36(2)	25(2)	3.0(19)	11.0(19)	-4.3(19)

Table S5. Bond Lengths for 8b.

**Atom**	**Atom**	**Length/Å**	**Atom**	**Atom**	**Length/Å**
Br1	C14	1.909(4)	O7	C15	1.435(5)
O1	C16	1.426(5)	C8	C12	1.382(5)
O1	C22	1.436(5)	C8	C13	1.382(5)
O2	C11	1.426(4)	C9	C10	1.194(5)
C3	C12	1.361(6)	C9	C16	1.473(5)
C3	C17	1.398(6)	C10	C11	1.480(5)
O5	C8	1.396(4)	C11	C13	1.520(5)
O5	C15	1.433(5)	C13	C14	1.406(5)
O6	C19	1.453(5)	C14	C17	1.384(5)
O6	C20	1.442(5)	C19	C20	1.444(6)
O7	C12	1.378(5)	C20	C22	1.484(6)

Table S6. Bond Angles for 5b**.**

Atom	Atom	Atom	Angle/°	Atom	Atom	Atom	Angle/°
C16	O1	C22	110.6(3)	O7	C12	C8	109.8(3)
C12	C3	C17	116.8(4)	C8	C13	C11	122.2(3)
C8	O5	C15	105.0(3)	C8	C13	C14	114.0(3)
C20	O6	C19	59.8(3)	C14	C13	C11	123.8(3)
C12	O7	C15	106.0(3)	C13	C14	Br1	120.4(3)
C12	C8	O5	110.1(3)	C17	C14	Br1	116.4(3)
C13	C8	O5	126.5(3)	C17	C14	C13	123.2(3)
C13	C8	C12	123.3(4)	O5	C15	O7	108.6(3)
C10	C9	C16	175.7(4)	O1	C16	C9	111.9(3)
C9	C10	C11	178.0(4)	C14	C17	C3	120.6(4)
O2	C11	C10	112.6(3)	C20	C19	O6	59.7(3)
O2	C11	C13	112.6(3)	O6	C20	C19	60.5(3)
C10	C11	C13	111.5(3)	O6	C20	C22	116.7(4)
C3	C12	O7	128.1(4)	C19	C20	C22	120.8(4)
C3	C12	C8	122.1(4)	O1	C22	C20	109.1(3)

Table S7. Hydrogen Atom Coordinates (Å×10^4) and Isotropic Displacement Parameters (Å^2×10^3) for 5b.

Atom	*x*	*y*	*z*	U(eq)
H2	1822	6648	791	33
H3	4050	5613	7886	30
H11	2497	5401	912	21
H15A	4989	7513	5801	38
H15B	6918	7217	6227	38
H16A	8589	5847	1686	27
H16B	7643	6336	175	27
H17	2333	4826	5883	27
H19A	11731	6667	7426	33
H19B	11264	7405	7818	33
H20	9060	7512	5033	45
H22A	10209	6248	4492	37
H22B	8233	6389	4300	37

Experimental

Single crystals of $C_{28}H_{28}O_{11}Br$ were dertermined**.** A suitable crystal was selected and the data was collected on a Xcalibur, Ruby, Gemini ultra diffractometer. The crystal was kept at 108.0(3) K during data collection. Using Olex2 [1], the structure was solved with the ShelXS [2] structure solution program using Direct Methods and refined with the ShelXL [3] refinement package using Least Squares minimisation.

1. O. V. Dolomanov, L. J. Bourhis, R. J. Gildea, J. A. K. Howard and H. Puschmann, OLEX2: a complete structure solution, refinement and analysis program. J. Appl. Cryst. (2009). 42, 339-341.
2. SHELXS, G.M. Sheldrick, Acta Cryst. (2008). A64, 112-122
3. SHELXL, G.M. Sheldrick, Acta Cryst. (2008). A64, 112-122

Crystal structure determination of 5b

Crystal Data for $C_{28}H_{28}O_{11}Br$ (M =620.41): monoclinic, space group $P2_1/c$ (no. 14), a = 8.15581(18) Å, b = 20.5484(3) Å, c = 8.7485(2) Å, β = 115.872(3)°, V = 1319.20(5) Å^3, Z = 2, T = 108.0(3) K, μ(Cu Kα) = 2.680 mm^{-1}, $Dcalc$ = 1.562 g/mm^3, 8567 reflections measured ($8.6 \leq 2\Theta \leq 124.76$), 2097 unique ($R_{int}$ = 0.0243) which were used in all calculations. The final R_1 was 0.0368 (>2sigma(I)) and wR_2 was 0.0783 (all data).
